# Structural reorganization of cylindrical nanoparticles triggered by polylactide stereocomplexation

**DOI:** 10.1038/ncomms6746

**Published:** 2014-12-17

**Authors:** Liang Sun, Anaïs Pitto-Barry, Nigel Kirby, Tara L. Schiller, Ana M. Sanchez, M. Adam Dyson, Jeremy Sloan, Neil R. Wilson, Rachel K. O’Reilly, Andrew P. Dove

**Affiliations:** 1 Department of Chemistry, University of Warwick, Coventry CV4 7AL, UK; 2 Australian Synchrotron, 800 Blackburn Road, Clayton, Victoria 3168, Australia; 3 Department of Materials Engineering, Monash University, Clayton, Melbourne, Victoria 3800, Australia; 4 School of Science and Technology, University of New England, New England, New South Wales 2351 Australia; 5 Department of Physics, University of Warwick, Coventry CV4 7AL, UK

## Abstract

Co-crystallization of polymers with different configurations/tacticities provides access to materials with enhanced performance. The stereocomplexation of isotactic poly(*L*-lactide) and poly(*D*-lactide) has led to improved properties compared with each homochiral material. Herein, we report the preparation of stereocomplex micelles from a mixture of poly(*L*-lactide)-*b*-poly(acrylic acid) and poly(*D*-lactide)-*b*-poly(acrylic acid) diblock copolymers in water *via* crystallization-driven self-assembly. During the formation of these stereocomplex micelles, an unexpected morphological transition results in the formation of dense crystalline spherical micelles rather than cylinders. Furthermore, mixture of cylinders with opposite homochirality in either THF/H_2_O mixtures or in pure water at 65 °C leads to disassembly into stereocomplexed spherical micelles. Similarly, a transition is also observed in a related PEO-*b*-PLLA/PEO-*b*-PDLA system, demonstrating wider applicability. This new mechanism for morphological reorganization, through competitive crystallization and stereocomplexation and without the requirement for an external stimulus, allows for new opportunities in controlled release and delivery applications.

## Abstract (web summary)

A polymer stereocomplex can possess quite different properties to its constituent homopolymers. Here, the authors prepare stereocomplex micelles of amphiphilic block-copolymers via crystallization-driven self-assembly, and observe a change from cylindrical to mixed spherical micelle morphology.

## Graphical abstract

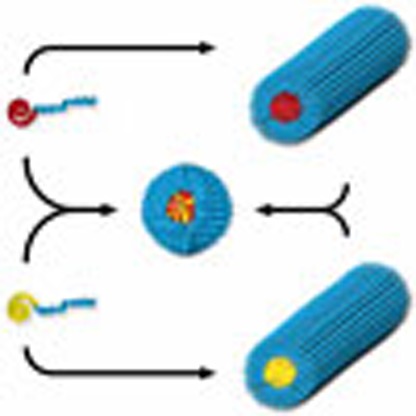

Polylactide has aroused great interest in the biomedical and pharmaceutical fields in the past few decades owing to its outstanding biodegradability, biocompatibility and low toxicity^1^,^2^,^3^,^4^,^5^. The cyclic lactide monomer presents two stereocenters and thus provides three stereoisomeric forms: *L*-lactide, *D*-lactide and *meso*-lactide. The polymerization of *L*-lactide or *D*-lactide leads to the formation of isotatic poly(*L*-lactide) (PLLA) and poly(*D*-lactide) (PDLA), which can co-crystallize to form the stereocomplex between the two polymer chains with opposite configurations. The stereocomplexation of polylactide was reported in 1987 by Ikada *et al*.^6^ who used differential scanning calorimetry and wide-angle X-ray diffraction (WAXD) to study the stereocomplexation of different ratios of PLLA and PDLA blends. It has been reported that the stereocomplex polylactide crystallites are stabilized by the CH_3_···O=C hydrogen-bonding interaction between the two opposite homochiral polymer chains, allowing a more compact conformation and a more dense polymeric packing than homochiral crystallites^7^. Stereocomplexation is of great interest in materials science, as the stereocomplex formed between the left-handed PLLA and right-handed PDLA polymeric helices has been shown to possess improved mechanical and thermal properties when compared with the homochiral polymers^2^,^6^,^8^,^9^. Beyond the stereocomplex formation in homopolymer blends, the self-assembly of polylactide (PLA)-containing block copolymers in selective solvents has opened up substantial opportunities to fabricate various nanoparticles with optimized properties^10^,^11^,^12^,^13^,^14^,^15^,^16^,^17^. For instance, stereocomplexation of poly(*L*-lactide)-*b*-poly(ethylene glycol) (PLLA-*b*-PEG) and poly(*D*-lactide)-*b*-poly(ethylene glycol) (PDLA-*b*-PEG) diblock copolymers resulted in enhanced stability of the resultant micellar constructs^12^. Furthermore, the stereocomplex micelles prepared from Y-shaped PEG-*b*-PLLA-*b*-PLLA and PEG-*b*-PDLA-*b*-PDLA miktoarm copolymers showed high loadings of paclitaxel and enhanced stability^14^. The stereocomplexation of PLLA and PDLA has also enabled access to a wide variety of nanoparticle morphologies^15^,^16^. Another approach, in which the stability has been predicted by theoretical simulations^18^,^19^, uses a mixed micelle approach where block copolymers with complementary blocks that have selective interactions between either the core or shell-forming blocks enable the access to a range of morphologies^20^,^21^. This concept has also been applied to PLA-containing materials, for example, Hedrick and co-workers obtained patchy polymeric micelles from the mixture of poly(*L*-lactide)-*b*-poly(*N*-isopropylacrylamide) (PLLA-*b*-PNIPAAM) and PDLA-*b*-PEG diblock copolymers^13^, whereas Bouteiller and researchers observed cylindrical micelles by mixing poly(*L*-lactide)-*b*-poly(ε-caprolactone) and poly(*D*-lactide)-*b*-poly(ε-caprolactone) in tetrahydrofuran (THF)^10^,^17^. In our previous studies^22^,^23^,^24^,^25^, we have shown PLLA-*b*-poly(acrylic acid) or PDLA-*b*-PAA diblock copolymers with a large hydrophilic weight fraction (82%) can assemble into homochiral cylindrical micelles through a crystallization-driven self-assembly (CDSA) process in solution above the glass transition temperature of the PLA block (typically 55–60 °C)^22^. These cylindrical micelles were also able to load hydrophobic dyes inside their hollow cores, which were induced by the hydrogen-bonding interaction between PLLA core and PAA corona during the slow-drying process^23^.

Herein, we report our studies into the application of stereocomplexation in CDSA processes. In an attempt to form more stable cylinders through stereocomplexation, we explore the assembly of a mixture of PLLA-*b*-PAA and PDLA-*b*-PAA diblock copolymers, under the same assembly conditions and surprisingly observed the formation of spherical micelles with stereocomplex cores. Interestingly, when mixed under CDSA conditions, the two homochiral cylinders undergo a morphological transition into spherical micelles with a PLA stereocomplex core. This transformation, in which cylinders transform into spheres, is the opposite of what is usually observed and as such is a rare observation; this is more so given that the core of the micelles in this system is highly crystalline^26^. This stereocomplex-induced reorganization is also demonstrated for a biocompatible PEG-*b*-PLA block copolymer system, as well as through the addition of a unimer solution to a solution of cylinders of opposite homochirality. This new mechanism of structural reorganization provides a potential new trigger for controlled/targeted drug release in polymeric vehicles.

## Results

### Synthesis of the precursor diblock copolymers

Diblock copolymers used in this study were achieved by a combination of ring-opening polymerization (ROP) of either *L*- or *D*-lactide with a highly selective metal-free thiourea/(-)-sparteine co-catalyst system and reversible addition-fragmentation chain transfer polymerization of tetrahydropyran acrylate (THPA) monomer from a dual-headed initiator as reported previously^22^,^25^. The compositions of the precursor diblock copolymers were determined by ^1^H NMR spectroscopy, resulting in PLLA_31_-*b*-PTHPA_332_ (1) and PDLA_28_-*b*-PTHPA_315_ (2). All the polymers used in this study possess low dispersities (*Đ*_M_<1.20), determined by size exclusion chromatography (SEC) analysis. Their characterization details are available in the Supplementary Information (Supplementary Figs 1–7 and Supplementary Table 1).

### Self-assembly of diblock copolymers

The exploration of the stereocomplex formation of PLLA-*b*-PAA and PDLA-*b*-PAA was conducted using the CDSA conditions reported previously^22^,^25^ but with a mixture of the two homochiral diblock copolymers. Specifically, 1 and 2 were mixed in an equal mass ratio in a vial before addition of a mixture of THF and H_2_O (*v*_THF_/*v*_H2O_=20/80), giving the final concentration of diblock copolymers of 20 mg ml^−1^. Acetic acid was added into the mixture to promote the hydrolysis of PTHPA into PAA and hence allow the formation of amphiphilic diblock copolymers. The sealed vial was pierced with a needle on top to allow the evaporation of THF during the self-assembly process. The temperature of the self-assembly was 65 °C, which is above the *T*_g_ of polylactide^27^, to allow the PLA blocks to move more freely and thus promote the co-crystallization of PLLA and PDLA. We have previously demonstrated that the PTHPA block can be completely converted into PAA within the first 2 h of the self-assembly^24^.

**Figure 1:**
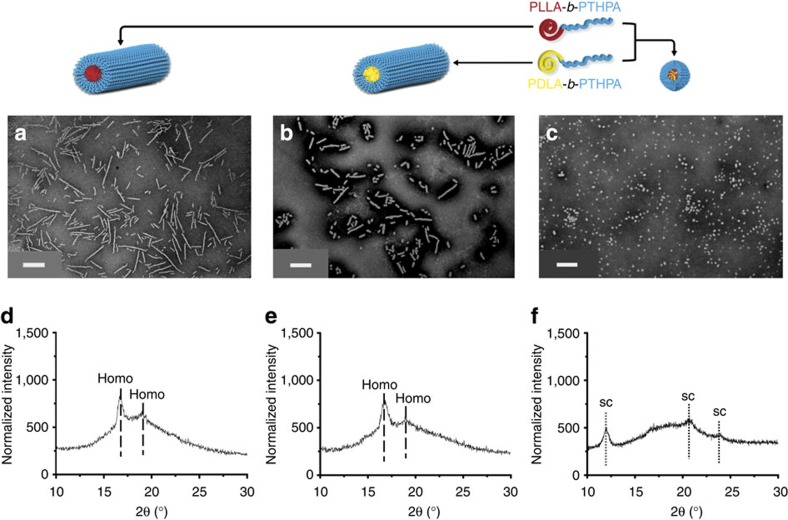
Characterization data of the nanostructures obtained from the self-assembly of diblock copolymers 1 or/and 2. Assembly performed in THF/H_2_O (20/80 v/v) at 65 °C. (**a**) TEM image of cylindrical micelles achieved from the self-assembly of the L-enantiomer 1; (**b**) TEM image of cylindrical micelles achieved from the self-assembly of the D-enantiomer 2; (**c**) TEM image of spherical micelles obtained from the mixture of the L and D -enantiomers 1 and 2; (**d**,**e**) The homochiral cylinders showing Bragg peaks at 2*θ* values of 16.6° and 19.2° from WAXD diffractograms; (**f**) Stereocomplex spheres gave Bragg peaks at 2*θ* values of 12°, 20.6°, 23.8°. All assemblies were run for 30 h before analysis. TEM samples were prepared by slow drying and negatively stained using phosphotungstic acid (PTA). Scale bars=500 nm.

After 30 h of self-assembly, spherical micelles (*D*_n_=50±7 nm) were observed using transmission electron microscopy (TEM) (Fig. 1c). The observed spherical morphology was not expected as under the same self-assembly conditions, the homochiral PLLA-*b*-PAA (or PDLA-*b*-PAA) diblock copolymer leads to the formation of a cylindrical morphology (Fig. 1a,b)^22^,^23^,^24^,^25^. We had hypothesized that a more stable cylindrical structure would form as a result of the stereocomplexation of the diblock copolymers bearing PLLA or PDLA blocks as observed in a related system by Boutellier and co-workers^10^,^17^. WAXD was used to confirm the successful co-crystallization of the two enantiomeric PLA blocks in the core domain. An intense Bragg peak at a 2*θ* value of 12° (which belongs to the (001) plane) definitively proved the crystalline stereocomplex formation (Fig. 1f), whereas the two homochiral cylinders obtained from 1 and 2, respectively, showed a completely different crystalline Bragg peak at a 2*θ* value of 16.6° (which belongs to the (110)/(200) planes) (Fig. 1d,e)^2^,^6^.

**Figure 2:**
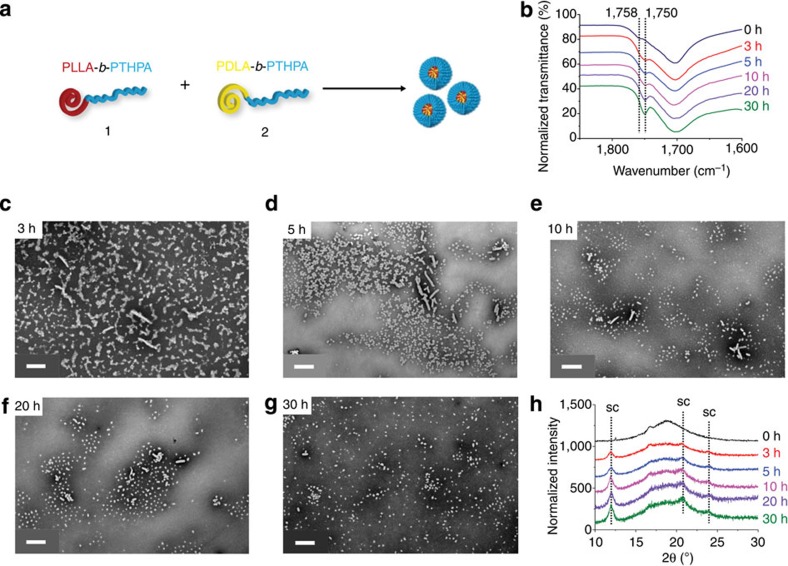
Characterization of the formation of the stereocomplex spherical micelles from the homochiral polymers 1 and 2. Studies conducted in THF/H_2_O (20/80 v/v) at 65 °C as a function of time. (**a**) Cartoon illustration of the formation of stereocomplex spherical micelles from 1 and 2. (**b**) FT-IR spectra of dried nanoparticles revealed that the wavenumber of carbonyl vibrational stretch of polylactide shifted from 1,758 to 1,750 cm^−1^ over time. TEM images: (**c**) 3 h after starting the self-assembly; (**d**) after 5 h; (**e**) after 10 h; (**f**) after 20 h and (**g**) after 30 h. (**h**) WAXD diffractograms showed that the Bragg peak for stereocomplex formation between 1 and 2 increased over time. Cylindrical micelles obtained from the homochiral diblock copolymers 1 or 2 were found to decrease in length during the self-assembly (**c**–**g**). TEM samples were prepared by slow drying and negatively stained using PTA. Scale bars=500 nm.

The self-assembly process for the formation of stereocomplex spheres was further investigated by fourier transfom-infrared (FT-IR) spectroscopy using dried nanoparticle samples (Fig. 2b). The vibrational stretch of the carbonyl group of polylactide was found to shift to a lower wavenumber (from 1,758 to 1,750 cm^−1^) after 3 h (Fig. 2b). Such a shift is attributed to the arrangement of the polylactide chains from a disordered state to an ordered one throughout the stereocomplex formation^10^,^17^. By using WAXD (Fig. 2h), an obvious stereocomplex Bragg peak at a 2*θ* value of 12° is observed after 3 h, which indicates a fast formation of the stereocomplex micelles. Prolonged assembly under these conditions promoted significantly the stereocomplexation of 1 and 2 as well as the slight increase in diameter of these spheres (from 42±7 nm in Fig. 2d to 50±7 nm in Fig. 2g, respectively). A careful study of the TEM images during the self-assembly process (Fig. 2c–g) revealed an interesting phenomenon: a number of cylinders existed at the beginning of the self-assembly, but their lengths gradually decreased, and as such, the cylindrical morphology almost disappeared after 30 h. As the cylinders are believed to only self-assemble from the homochiral diblock copolymer 1 or 2 but not from the stereocomplex, we propose that the crystallization process is fast and leads to the resolution of the mixture to *D* and *L* homochiral cylinders, which over time undergo a morphological transition into stereocomplex spheres. Such a morphological transition is unusual and only one example has been previously reported^26^. Indeed, this observation is in contrast to that observed by Boutellier and coworkers^10^,^17^ in which mixtures of poly(ε-caprolactone)-*b*-PLLA and poly(ε-caprolactone)-*b*-PDLA copolymers were self-assembled into cylinders in THF. These differences in behaviour are attributed to an absence of selective solvation and more similar block volume ratios than applied in this work.

### Stereocomplexation triggered morphological transitions

**Figure 3:**
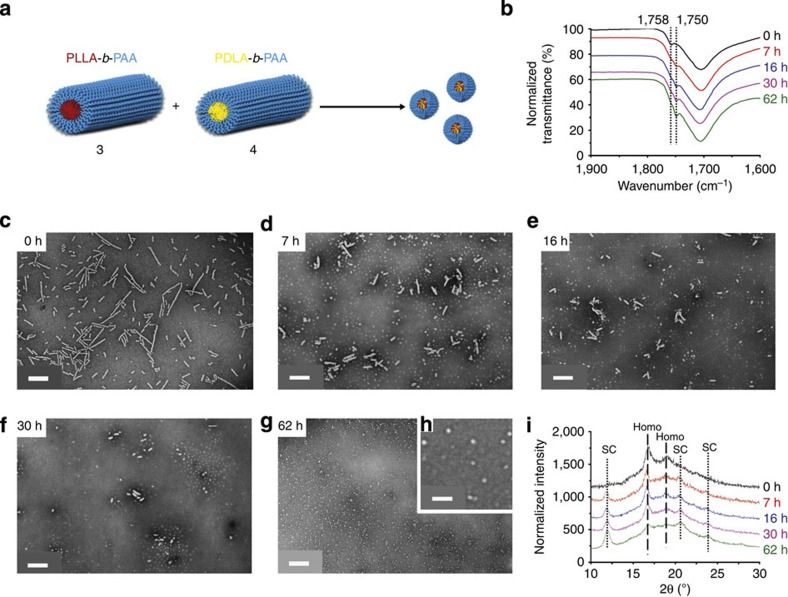
Morphological transition and the changes to the crystallinity on mixing of two homochiral cylinders 3 and 4. Conducted at 65 °C with the addition of fresh THF (final solvent composition THF/H_2_O 20/80 v/v). (**a**) Cartoon illustration showing the morphological transition from homochiral cylinders 3 and 4 to stereocomplex spheres. Scale bar=500 nm (**b**) FT-IR spectra of dried nanoparticles, which reveal the wavenumber of carbonyl group vibration of poly(lactide) shifted from 1,758 to 1,750 cm^−1^ over time. Scale bar=500 nm. (**c**–**g**) TEM images, which illustrate the length of the cylindrical micelles decreased while the population of spherical micelles increased over time. Scale bars=500 nm. (**h**) TEM expansion of 62 h time point. Scale bar=100 nm. (**i**) WAXD diffractograms illustrating that the intensity of stereocomplex Bragg peak at a 2*θ* value of 12° increased gradually, whereas the intensity of homochiral Bragg peak at a 2*θ* value of 16.6° decreased significantly over time. TEM samples were prepared by slow drying and negatively stained using PTA.

**Figure 4:**
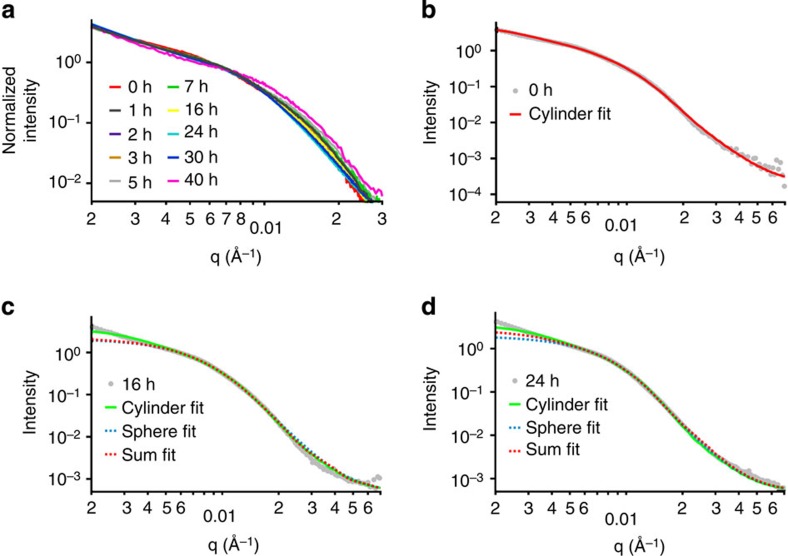
SAXS experimental profiles and fittings of the morphological transition from the two homochiral cylinders 3 and 4 to stereocomplex spheres. Conducted at 65 °C with the addition of fresh THF at the beginning of the self-assembly. (**a**) SAXS experimental profiles during the entire self-assembly process: a change of morphology is observed, with a decrease of the radius of the morphology as the curve inflexions move to higher *q*-values with time. (**b**–**d**) Fitting of some experimental profiles with different models: cylinder (**b**) and a linear combination of sphere and cylinder (**c**,**d**), see also Supplementary Table 2.

To explore the morphological transition in more detail and demonstrate its potential utility, separate solutions of homochiral cylinders 3 (*L*_n_=194 nm, *L*_w_/*L*_n_=1.22, *W*_n_=53±6 nm) and 4 (*L*_n_=188 nm, *L*_w_/*L*_n_=1.21, *W*_n_=54±7 nm) were prepared from diblock copolymers 1 and 2, respectively, (Fig. 1a,b) under CDSA conditions for 30 h as described above. Subsequently, 1 ml (around 17 mg ml^−1^) of each cylinder solution (in pure water) was mixed together with an additional 0.5 ml of THF to the cylinder mixture to mimic the starting self-assembly conditions described above (*v*_THF_/*v*_H2O_=20/80). The solution was then heated at 65 °C with a needle added through the cap to allow the THF to evaporate (Fig. 3a). By using such an approach, we observed a marked decrease in cylinder lengths and increase in the population of spherical micelles by TEM (Fig. 3c–g), which demonstrates the ability to trigger the morphological transition through the onset of stereocomplexation. Analysis by dynamic light scattering (DLS) also illustrated the decrease in the hydrodynamic diameters of the nanoparticles over time (Supplementary Fig. 8). *In situ* synchrotron small-angle X-ray scattering (SAXS) further confirmed the cylinder-to-sphere transition (Fig. 4). A comparison of the parameters obtained from the fitted SAXS curves (Supplementary Table 2 and Supplementary Fig. 9) revealed the presence of an increasing number of spheres along with a decreasing dimension of cylinders over time. These observations were in accordance with TEM and DLS results.

FT-IR spectroscopic analysis was performed on samples taken throughout the cylinder-to-sphere transition to prove that the stereocomplex formation had occurred (Fig. 3b). A lower wavenumber shift from 1,758 to 1,750 cm^−1^ was observed for the vibration of the carbonyl group of the polylactide chains, which suggests the successful formation of stereocomplex micelles. By using WAXD (Fig. 3i), a significant decrease in the intensity of the homochiral peak at a 2*θ* value of 16.6° and a gradual intensity increase of the stereocomplex peak at 2*θ* value of 12° were observed. Importantly, the same morphological transition from homochiral cylinders to stereocomplex spheres was also observed when 3 and 4 were mixed and heated without the addition of fresh THF as evidenced by TEM, albeit over a longer time period (Supplementary Figs 10c–10g), DLS (Supplementary Fig. 11), SAXS (Supplementary Fig. 12 and Supplementary Table 3), FT-IR (Supplementary Fig. 10b) and WAXD (Supplementary Fig. 10h). Furthermore, following complete removal of THF from the system by freeze-drying the homochiral cylinders, the morpohological transition was still observed by the expected change in particle morphology by TEM and DLS (Supplementary Fig. 13). We propose that the stereocomplexation is hindered without the presence of a good solvent for polylactide (such as THF), which assists in chain folding and the co-crystallization in the core domain. The dimensions of the stereocomplex spheres synthesized from unimers (*D*_n_=49.7 nm, Fig. 1c) are much larger (more than twice) than those of stereocomplex spheres achieved from disassembly of cylinders (*D*_n_=22±3 nm for Fig. 3g, *D*_n_=20±5 nm for Supplementary Fig. 10g), as indicated by TEM analysis. As the stereocomplex core and PAA corona are both visible after staining with phosphotungstic acid (PTA), the crystalline stereocomplex core domain appears even smaller than that observed using negative staining.

**Figure 5:**
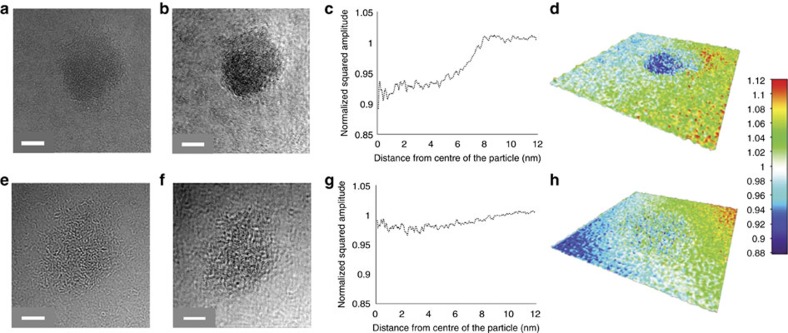
Microscopy analysis of stereocomplex and amporphous spherical micelles. (**a**–**d**) A stereocomplex spherical micelle (obtained from the mixture of homochiral cylinders 3 and 4) and (**e**–**h**) an amorphous spherical micelle (obtained from the self-assembly of 1). (**a**) Near focus aberration-corrected microscope image and (**b**) EWR-squared amplitude image of a stereocomplex sphere; (**e**) Near focus aberration-corrected microscope image and (**f**) EWR-squared amplitude image of an amorphous sphere. All of the images were obtained on graphene oxide supports. The increased density of the stereocomplex sphere relative to the amorphous sphere can be seen in the stronger contrast in the squared amplitude image, this is shown quantitatively in panels **c** and **g** (for the stereocomplex micelle and amorphous micelle, respectively), which plots the radially averaged profile of the squared amplitude from the middle of each sphere to the GO support. Panels **d** and **h** show the surface plots of the EWR-squared amplitude images demonstrate the higher density of the stereocomplex micelle. Scale bars=5 nm.

To further compare the stereocomplex spherical micelle (Fig. 5a, the same sample shown in Fig. 3g) and amorphous spherical micelle (Fig. 5e, obtained from the self-assembly of 1, the preparation method is given in Methods, WAXD in Supplementary Fig 14), we used an aberration-corrected high resolution TEM at near focus condition using graphene oxide (GO) supports, which are nearly electron transparent^28^. Exit wave reconstruction (EWR) enabled a higher contrast and higher resolution image to be obtained from a focal series of TEM images, despite the low contrast of predominantly carbon-based materials, and thus provided more structural detail for the macromolecular nanostructures^29^. EWR gives a phase image and an amplitude image; macromolecular nanostructures exhibit mainly phase contrast in the TEM and so are clearly resolved in the phase images. The square of the amplitude image (the intensity) gives contrast, which is roughly related to the mass thickness of the macromolecular sample. By using EWR on unstained samples, a much clearer spherical shape was observed for both stereocomplex and amorphous micelles in the phase (Supplementary Fig. 15). The squared amplitude images from EWR (Fig. 5b,f) revealed that the stereocomplex spherical micelle possessed a higher density than the amorphous spherical micelle, with the intensity relative to the background decreased by 8% for the former but only 3% for the latter (Fig. 5c,g) (decreased intensity is due to increased scattering of the electron beam and hence implies increased mass thickness). The surface plots of EWR squared amplitude images (Fig. 5d,h) demonstrate the higher density of the stereocomplex micelle and thus provide further evidence of its altered physical state.

To prove that the effects observed were a consequence of the onset of stereocomplexation, two control experiments were conducted using identical self-assembly conditions as described above, to monitor the changes in the homochiral cylinder 3 only. The single enantiomer, homochiral cylindrical micelles were heated at 65 °C for an extended time at a high concentration (17 mg ml^−1^). With no addition of fresh THF, the length of these cylinders stayed approximately the same even after 120 h of heating, although some aggregation was observed by TEM analysis (Supplementary Figs 16a–16c). This observation was supported by SAXS analysis (Supplementary Fig. 17, Supplementary Table 4), which also indicated unchanged cylinder length as well as some aggregation after excessive heating. Moreover, no obvious changes in the intensity of the Bragg peak at a 2*θ* value of 16.6° were observed from WAXD diffractograms over time (Supplementary Fig. 16d), again indicating the high stability of these cylinders. Interestingly, when the cylinder solution was exposed to fresh THF (*v*_THF_/*v*_H2O_=20/80) at 65 °C, with a needle through the seal of the vial, the cylinder length decreased rapidly within the first few hours but then large lamellar particles were formed after 16 h, as observed by TEM analysis (Supplementary Figs 18a–18e). The formation of platelets rather than cylinders on addition of a common solvent has been detected previously for crystalline-coil systems^30^. These trends were further confirmed by SAXS with an observed increase in the population of lamellar particles compared with the population of cylinders over the heating process (Supplementary Fig. 19 and Supplementary Table 5). WAXD diffractograms of this system also indicated that the intensity of the homochiral peak at the 2*θ* value of 16.6° decreased after the addition of fresh THF into the cylinder solution but recovered after another 30 h of heating under the self-assembly conditions (Supplementary Fig. 18f). The lack of cylinder-to-sphere transition was confirmed by TEM and SAXS during these two control experiments.

The stereocomplexation-triggered morphological transition was also investigated for a fully biocompatible PLA system (PLLA-*b*-PEG and PDLA-*b*-PEG (Supplementary Figs 20 and 21)), which was synthesized as we have reported previously^31^. Over a similar timeframe, the PLA-*b*-PEG copolymer system also demonstrated a transition from crystalline cylinders to stereocomplex spheres. We suggest that this new mechanism for nanostructure reorganization, which is triggered by stereocomplexation, highlights the potential utility of these PLA materials in controlled release or delivery applications across a wide range of applications.

### Explanation of the morphological transition

The exact mechanism of cylinder growth in this and related systems is not well understood, with two possible mechanisms suggested by Eisenberg *et al*.^32^ in the late 1990s: the unimer-exchange theory, in which cylindrical nanoparticles are proposed to grow by an exchange of unimers between particles, and the adhesive fusion theory, in which it is proposed that micelles collide together and adhere to one another to form cylinders. Although much evidence to date has supported the adhesive fusion mechanism^33^,^34^, several in-depth studies from the Manners, Winnik and Schmalz groups have suggested that a unimer-exchange mechanism occurs to some extent when epitaxial growth of crystalline-core cylinders occurs at elevated temperature (in a selective solvent) or at room temperature (on addition of a common solvent to the selective solvent)^35^,^36^,^37^,^38^,^39^. In the present study, the morphological transition from homochiral cylinders into stereocomplex spheres is somewhat unexpected given that under thermodynamic control, mixed micelle formation between two polymers of such structural similarity would be expected to retain the cylindrical morphology. The stereocomplexation driving force that exists in this system changes the crystallization behaviour of the core-forming blocks and hence is able to drive this morphology switch. Importantly, these results are highly consistent with the existence of unimers in solution under assembly/disassembly conditions. When the cylinder solutions were mixed, stereocomplex micelles formed gradually from these dynamic unimers with both the *L* and *D* unimers locked into the stereocomplex micelles. Only by being present as unimers in solution could the components of different stereochemistry interact sufficiently to form stereocomplex micelles that display a higher stability owing to the improved packing and strong interactions of the helical chains of opposite configurations^2^,^6^,^7^,^40^. In an adhesive fusion process, homochiral cylinders would be expected to form when 3 and 4 were mixed, however, this phenomenon was not observed. Such a unimer-exchange process was further confirmed when the addition of *D**-*unimers (dissolved in THF) into aqueous solution of *L**-*cylinders (3) led to the disassembly of the latter and the formation of the stereocomplex spherical micelles (Supplementary Fig. 22). Although the disassembly is accelerated by the addition of a common solvent for both blocks, its observation provides a potential new trigger to induce a morphological transition or release from nanoscale particles in a completely aqueous system.

In summary, we have demonstrated that stereocomplexation can be used to trigger the reorganization of homochiral PLA-containing cylindrical micelles to form small stereocomplex spherical micelles, in contrast to that usually observed in CDSA systems. This morphology transition has been monitored by TEM, DLS, *in situ* synchrotron SAXS, WAXD and FT-IR analysis. Such a morphological transition was not observed when enantiopure PLLA-*b*-PAA cylinders were exposed to the same self-assembly conditions without the presence of the opposite enantiopure PDLA-*b*-PAA cylinders. Both the mechanism of the cylinder-to-sphere transition in this system and the homochiral cylinder growth were confirmed to occur by a unimer-exchange process. We propose that this fundamental study provides a deeper understanding of the balance of self-assembly forces in such PLA systems and suggest that this stereocomplexation-triggered reorganization may have potential applications in delivery/controlled release.

## Methods

### Materials

Chemicals and solvents were used as purchased from Aldrich, Acros, Fluka, Fisher Chemical, Alfa Aesar or VWR. *L*-Lactide and *D*-lactide monomers were kindly donated by Corbion-Purac and were passed through a silica plug with dichloromethane (CH_2_Cl_2_) as eluent to remove impurities and then dried over 3 Å molecular sieves in CHCl_3_. The lactide monomers were further purified by recrystallization in toluene before being stored into a glovebox with inert atmosphere. (-)-Sparteine was dried over CaH_2_ before use and 1-(3,5-bis(trifluoromethyl)phenyl)-3-cyclohexyl-thiourea was prepared and purified as reported^41^. THPA was synthesized and purified as described previously^42^. 2,2-azobis(isobutyronitrile) was recrystallized from methanol and stored at 4 °C. PEG was purchased from Iris Biotech and dried in a desiccator under vacuum over P_2_O_5_.

### Instrumentation

^1^H NMR spectra were recorded on Bruker spectrometers operating at a frequency of 300 or 400 MHz in CDCl_3_. The chemical shifts are given in ppm with tetramethylsilane as internal reference. SEC was performed on an Agilent 1,260 Infinity Multi-Detector SEC instrument equipped with refractive index and ultraviolet detectors with THF and 2% triethyl amine as eluent at a flow rate of 1 ml  min^−1^. SEC data were calibrated by Cirrus GPC software with poly(styrene) standards.

FT-IR spectra were obtained using a Perkin Elmer Spectrum 100 FT-IR. Scans from 550 to 4,000 cm^−1^ were taken, and the spectra corrected for background absorbance, all curves were normalized.

Mass spectra were obtained by using a Bruker Ultraflex II Matrix-assisted laser desorption/ionization time of flight (MALDI-ToF) mass spectrometer. The MALDI-ToF samples were prepared as follows: *trans*-2-[3-(4-*tert*-butyl-phenyl)-2-methyl-2-propenylidene] malononitrile (DCTB) was used as a matrix while sodium trifluoroacetate was used as a cationization agent. Typically, DCTB (20 μl of a 40 mg ml^−1^ HPLC grade THF solution), samples (20 μl of a 1 mg ml^−1^ HPLC grade THF solution) and sodium trifluoroacetate (20 μl of a 0.1 mg ml^−1^ HPLC grade THF solution) were successively added into a small centrifuge tube and mixed by a vortex mixer. Traces of mixture were deposited on a MALDI-ToF plate followed by solvent evaporation. The samples were measured in reflectron ion mode and calibrated by SpheriCal (1,200~8,000 g  mol^−1^) standards.

The hydrodynamic diameter (*D*_h_) of different nanoparticles was determined by DLS. Typically, 0.25 mg ml^−1^ aqueous nanoparticle solution scattering was measured in a Malvern Zetasizer NanoS instrument equipped with a 4 mW He-Ne 633-nm laser module at 25 °C. Measurements were carried out at a detection angle of 173° (back scattering) and the data were further analyzed by Malvern DTS 6.20 software. *D*_h_ was calculated by fitting the apparent diffusion coefficient in the Stokes–Einstein equation *D*_h_=*kT*/(3π*ηD*_app_), where *k* is the Boltzmann constant, *T* is the temperature and *η* is the viscosity of the solvent. *D*_h_ only coincides to the real hydrodynamic diameter when the measured sample is a solution of monodispersed spherical particles as *D*_app_ equals the translational diffusion (*D*_t_). For cylindrical particles, owing to their anisotropy, the rotational diffusion is not negligible and contributes to the *D*_app_. Therefore, the *D*_h_ measured for the cylindrical micelles only has a relative value and provides polydispersity information to detect multiple populations.

TEM images on GO support (Fig. 5) were obtained by using an aberration-corrected JEOL JEM-ARM200F instrument operating at 80 kV with spherical aberration corrected tuned to ~+1 μm and images were recorded on a Gatan SC-1,000 Orius CCD camera. TEM samples were prepared using a freeze-drying method using GO-covered TEM grids that provide a thin support, which is almost electron transparent and gives excellent contrast^28^. Generally, one drop of each sample solution (5 μl of 0.1 mg ml^−1^) was added onto the GO grid and was frozen using liquid nitrogen for 15 s. The grid was then placed into a small vial inside a flask, which could be attached to a freeze drier. After lyophilization, the grid was collected for TEM analysis. Analysis was performed on a minimum of 20 particles of each type with 20 images in each focal series. The stained TEM images were obtained by using a JEOL 2000FX instrument operated at 200 kV. TEM samples were negatively stained by (phosphotungstic acid, 2 wt%) on formvar/carbon grid (300 Mesh, Cu, Elektron Technology UK LTD). Typically, formvar/carbon grids were cleaned by air plasma from a glow-discharge system (2 min, 20 mA), which also improved the hydrophilicity character of the grids. 20 μl of particle solution (0.25 mg ml^−1^) was added onto the grid and the solution was blotted away after 2 min and then left to air dry. Five microlitres of a 2 wt% PTA solution was then added on the grid to stain the particles and was blotted away after 30 s before air drying. TEM images were analyzed by ImageJ software, and 200 particles were counted for each sample to obtain the number-average length (*L*_n_) and to calculate weight-average length (*L*_w_), number-average width (*W*_n_) (for cylindrical micelles) and number-average diameter (*D*_n_) (for spherical micelles). *L*_n_, *L*_w_, *W*_n_ and *D*_n_ were calculated by using the following equations:


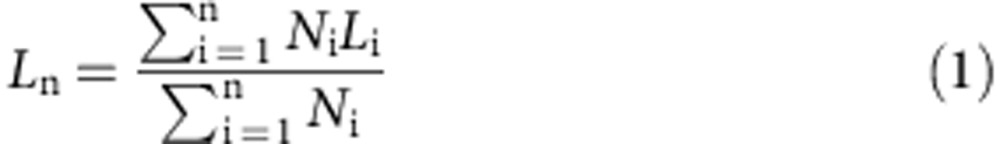



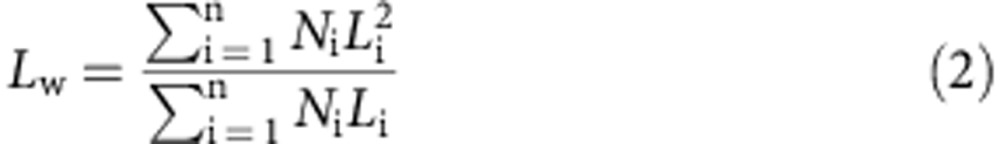



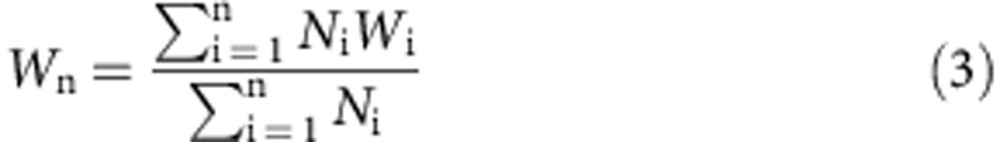



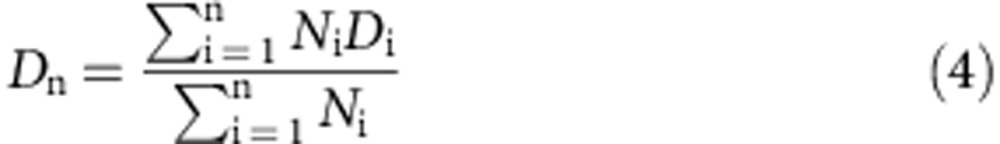


where *L*_i_ and *W*_i_ are the length and the width of each counted cylindrical micelle, whereas *D*_i_ is the diameter of each counted spherical micelle. *N*_i_ is the number of the cylindrical micelles with the length of *L*_i_ and the width of *W*_i_ or the number of the spherical micelles with the diameter of *D*_i_.

SAXS were carried on the SAXS/WAXS beamline at the Australian Synchrotron facility at a photon energy of 11 keV. The samples in solution were run using 1.5 mm diameter quartz capillaries. The reactions took place in heated vials, with the solutions circulating through the capillaries using a peristaltic pump. Such a setup allows living data to be collected *in situ*, which provides a real-time insight into the overall self-assembly process and the evolution of the morphologies. The measurements were collected at a sample to detector distance of 7.364 m to give a *q* range of 0.002–0.08 Å^−1^ (SAXS), where *q* is the scattering vector and is related to the scattering angle (2*θ*) and the photon wavelength (*λ*) by the following equation:


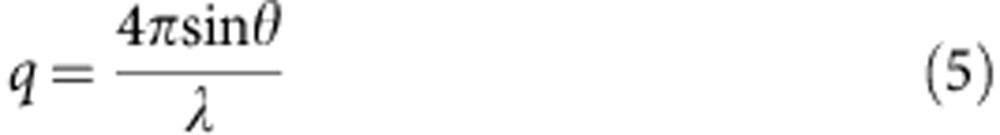


Data were processed using ScatterBrain for radial integration, normalization, absolute scaling and background subtraction: patterns were normalized to fixed transmitted flux using a quantitative beamstop detector; the scattering from a blank (H_2_O) was measured in the same capillary and was subtracted for each measurement; the two-dimensional SAXS images were converted in one-dimensional SAXS profile (*I*(*q*) *versus* *q*) by circular averaging, where *I*(*q*) is the scattering intensity. NIST SANS macros were used for SAXS data analysis using Igor Pro software^43^. The scattering length density of the solvent was calculated using the scattering length density calculator provided by the NIST Center for Neutron Research^44^. Limits for *q* range were applied for the fitting of SAXS data from 0.002 to 0.07 Å.

WAXD was performed on a Panalytical X’Pert Pro MPD equipped with a CuKα_1_ hybrid monochromator as the incident beam optics. Generally, ~30 mg of self-assembled freeze-dried particles were placed in a 10 mm sample holder, and standard powder 2*θ*−*θ* diffraction scans were carried out in the angular range from 2*θ* 10° to 30° at room temperature.

### Synthesis of dodecyl 4-(hydroxymethyl) benzyl carbonotrithioate

The dual-headed initiator was synthesized as reported previously^22^. Generally, dodecanethiol (1.29 g, 6.4 mmol), potassium phosphate (1.48 g, 7 mmol) and carbon disulphide (1.14 ml, 19 mmol) were added into acetone (200 ml). After stirring for 2 h at room temperature, 4-chloromethylbenzyl alcohol (1.00 g, 6.4 mmol) was added into the solution. The mixture was allowed to stir for another 24 h. Acetone was then removed *in vacuo* and the resultant solid was dissolved in CHCl_3_. The organic layer was washed against HCl (1 mol l^−1^, 200 ml × 1), distilled water (200 ml × 3) and brine (200 ml × 1) before being dried over magnesium sulfate. Solids were removed by filtration and the solution was concentrated *in vacuo* before purification through a silica column (*n*-hexane/ethyl acetate=60/40). The solvent was removed *in vacuo* to yield a yellow solid, which was dried over P_2_O_5_ in a dessicator for 2 days before being transferred into a glove box with inert atmosphere. ^1^H NMR (400 MHz, CDCl_3_) δ 7.40–7.28 (4H, m), 4.68 (2H, d, ^3^*J*_H-H_=5.0 Hz), 4.61 (2H, s), 3.37 (2H, t, ^3^*J*_H-H_=7.5 Hz), 1.77–1.58 (3H, m), 1.46–1.20 (18H, m), 0.88 (3H, t, ^3^*J*_H-H_=7.0 Hz); ^13^C NMR (150 MHz, CDCl_3_) δ 223.7, 140.4, 134.6, 129.5, 127.3, 65.0, 41.0, 37.1, 32-22, 14.1.

### ROP of *L*-lactide (*D*-lactide)

PLLA (or PDLA) was synthesized in a glove box under nitrogen atmosphere as reported previously^22^,^25^. Typically, for PLLA (DP=31), the dual-headed initiator (92.7 mg, 0.23 mmol) and (-)-sparteine (40.1 μl, 0.17 mmol) were combined in one vial with L-lactide (1.01 g, 6.98 mmol) and 1-(3,5-bis(trifluoromethyl)phenyl)-3-cyclohexyl-thiourea (129.2 mg, 0.35 mmol) in another. CHCl_3_ (4 and 6 ml, respectively) was then added to each of the vials before the two solutions were mixed and allowed to stir at room temperature for 3 h. The product was precipitated in *n*-hexane three times before filtration and drying *in vacuo* to yield a pale yellow solid. ^1^H NMR (400 MHz, CDCl_3_) δ 7.36–7.30 (4H, m), 5.40–5.30 (2H PLLA+2H, m), 4.61 (2H, s), 3.37 (2H, t, ^3^*J*_H-H_=7.5 Hz), 1.66–1.48 (6H PLLA+2H, m), 1.34–1.18 (18H, br), 0.88 (3H, t, ^3^*J*_H-H_=6.5 Hz).

### Synthesis of PLLA-*b*-PTHPA 1 (PDLA-*b*-PTHPA 2)

The representative procedure of the synthesis of diblock copolymer 1 is as follows^22^,^25^. Typically, PLLA_31_ macro-initiator (194.7 mg, 1 eq.) and THPA (2.5 g, 400 eq.) monomer were dissolved in CHCl_3_ (2.5 ml) and transferred into a dried ampoule before addition of azobis(isobutyronitrile) (65.8 μl of a 10 mg ml^−1^ CHCl_3_ solution). The solution was degassed by three freeze-pump-thaw cycles and sealed under argon and then placed in a 60 °C oil bath with stirring for 3.5 h. The product was precipitated into *n*-hexane for three times and dried *in vacuo* to give a pale yellow solid (76% conversion by ^1^H NMR spectroscopy) with a final yield of 98%. ^1^H NMR (400 MHz, CDCl_3_) δ 6.20–5.68 (1H PTHPA, br), 5.26–5.04 (2H PLLA, m), 3.96–3.58 (2H PTHPA, br), 2.66–2.24 (^1^H PTHPA, br), 2.18–1.36 (8H PTHPA & 6H PLLA, br m).

### ROP of *L*-lactide (*D*-lactide) from PEG(20k)

PEG-PLLA (or PEG-PDLA) was synthesized in a glove box under nitrogen atmosphere as reported previously^31^. Typically, for PLLA (DP=27), the PEG monomethyl ether initiator (300 mg, 0.015 mmol) and (-)-sparteine (2.67 μl, 0.012 mmol) were combined in one vial with *L*-lactide (67 mg, 0.465 mmol) and 1-(3,5-bis(trifluoromethyl)phenyl)-3-cyclohexyl-thiourea (8.6 mg, 0.023 mmol) in another. CH_2_Cl_2_ (1.6 and 0.3 ml, respectively) was then added to each of the vials before the two solutions were mixed and allowed to stir at room temperature for 31 h. The product was precipitated in cold diethyl ether three times before filtration and drying *in vacuo* to yield a pale white solid. ^1^H NMR (300 MHz, CDCl3) δ 5.21–5.13 (2H PLLA, q, ^3^*J*_H–H_=7.4 Hz), 3.64 (4H PEG, s), 3.38 (3H CH_3_O-PEG, s), 1.60–1.66 (6H PLLA, d, ^3^*J*_H–H_=6.2 Hz).

### Preparation of amorphous spheres from diblock copolymer 1

Diblock copolymer 1 (50 mg) was exposed to the identical CDSA conditions (*v*_THF_/*v*_H2O_=20/80, acetic acid (1 eq. per THPA block), evaporation of THF, 65 °C)^22^,^25^. After 2.5 h of self-assembly, as the morphology of the nanostructures was still a spherical structure (before the morphological transition into cylinder)^24^, the nanoparticle solution was quenched by cooling in liquid nitrogen and subsequently lyophilized. The spherical morphology was confirmed by using high resolution transmission electron microscope (Fig. 5e), and the amorphous core nature of these nanoparticles was evidenced by using WAXD (Supplementary Fig. S14).

### Self-assembly of 1 and 2 to realize stereocomplex particles

Identical self-assembly conditions that have been previously reported by the group to afford homochiral cylinders were utilized to obtain stereocomplex particles^22^,^25^. A 1:1 mixture of 1 (25 mg) and 2 (25 mg) were added in 0.5 ml THF to 2 ml of H_2_O (resistivity 18.2 MΩ·cm) (*v*/*v*=20/80) in a vial and sealed with a needle through the seal. Acetic acid (1 eq. per THPA block) was added into the mixture and the self-assembly was set up at 65 °C. After 30 h, the solution was quenched by cooling in liquid nitrogen and subsequently lyophilized. The freeze-dried particles were then redispersed in H_2_O (0.25 mg ml^−1^) at room temperature for TEM and DLS analysis.

### CDSA of 1 or 2

The identical CDSA conditions described above were used to prepare cylindrical micelle 3 or 4 from either diblock copolymer 1 or 2 as previously reported^22^,^25^. Generally, 0.5 ml THF and 2 ml H_2_O (resistivity 18.2 MΩ·cm) were added into 50 mg polymer inside a vial. Acetic acid (1 eq. per THPA block) was also added to the mixture. The vial was sealed with a needle through the seal and the mixture was allowed to stir at 65 °C for 30 h before being quenched in liquid nitrogen and subsequent lyophilization. The freeze-dried particles were then dissolved directly into H_2_O (0.25 mg ml^−1^) at room temperature for TEM and DLS analysis.

### Addition of PDLA-*b*-PAA unimers into PLLA-*b*-PAA cylinder 3

Diblock copolymer 2 (50 mg) was exposed to the identical CDSA conditions (*v*_THF_/*v*_H2O_=20/80, acetic acid (1 eq. per THPA block), evaporation of THF, 65 °C). After 2.5 h, as the PTHPA block was completely hydrolyzed into PAA block^24^, the solution was quenched by cooling in liquid nitrogen and subsequently lyophilized. The freeze-dried PDLA-*b*-PAA diblock copolymer (17 mg) was fully dissolved in THF (0.5 ml). DLS analysis confirmed that PDLA-*b*-PAA existed as unimer state in THF (Supplementary Fig. S22g). THF solution (0.5 ml) of PDLA-*b*-PAA unimers was then added into 2 ml aqueous solution of PLLA-*b*-PAA cylinder 3 (17 mg) in a vial. The vial was sealed with a needle through the seal and the mixture was allowed to stir at 65 °C. At different time intervals, the mixture was sampled and quenched by cooling in liquid nitrogen and subsequently lyophilized. The freeze-dried particles were then dissolved directly into H_2_O (0.25 mg ml^−1^) at room temperature for TEM and DLS analysis.

### Mixture of PLLA-*b*-PEG cylinder and PDLA-*b*-PEG cylinder

The homochiral PLLA-*b*-PEG cylinder and PDLA-*b*-PEG cylinder were prepared as previously reported by the group^31^. One millilitre of each cylinder solution (note: the cylinder solution still co-existed with a number of spherical micelles) was directly mixed (20 mg ml^−1^) before addition of 0.5 ml THF. The mixture was sealed with a needle through the seal and the mixture was allowed to stir at 65 °C. At different time intervals, the mixture was sampled and quenched by cooling in liquid nitrogen and subsequently lyophilized. The freeze-dried particles were analyzed by FT-IR and dissolved directly into H_2_O (0.25 mg ml^−1^) at room temperature for TEM analysis.

## Author contributions

L.S. designed the experiments, performed the experiments, analyzed and discussed the results. A.P.B. performed the SAXS analysis, some synthesis and characterization, discussed the results and commented on the paper. N.K. and T.S. designed the SAXS experiments and *in situ* analysis rig, assisted with SAXS analysis and commented on the paper. M.A.D. and A.S. performed the high-resolution microscopy and its EWR analysis and discussed the results and commented on the paper. N.R.W. and J.S. discussed the microscopy results and commented on the paper. A.P.D. and R.K.O.R. conceived and obtained funding for the project, designed experiments and discussed the results. The paper was written by L.S, A.P.D. and R.K.O.R.

## Additional information

**How to cite this article:** Sun, L. *et al*. Structural reorganization of cylindrical nanoparticles triggered by polylactide stereocomplexation. *Nat. Commun.* 5:5746 doi: 10.1038/ncomms6746 (2014).

## Supplementary Material

Supplementary Information Supplementary Figures 1-22 and Supplementary Tables 1-5.
