# Supplementary material for: Structural reorganization of cylindrical nanoparticles triggered by polylactide stereocomplexation
Source: Nat Commun. 2014 Dec 17;5:5746. doi: 10.1038/ncomms6746 (PMC4281646; doi:10.1038/ncomms6746)
Supplement: Supplementary Information — Supplementary Figures 1-22 and Supplementary Tables 1-5. [file ncomms6746-s1.pdf]

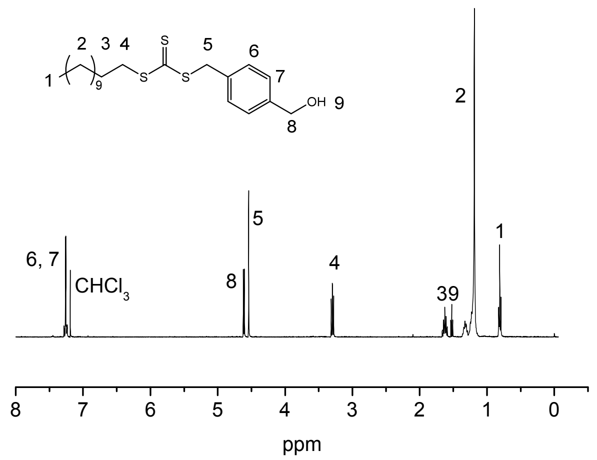

Supplementary Figure 1. ^{1}H NMR spectrum of dual-headed initiator dodecyl 4-(hydroxymethyl) benzyl carbonotrithioate (400 MHz, $CDCl_3$).

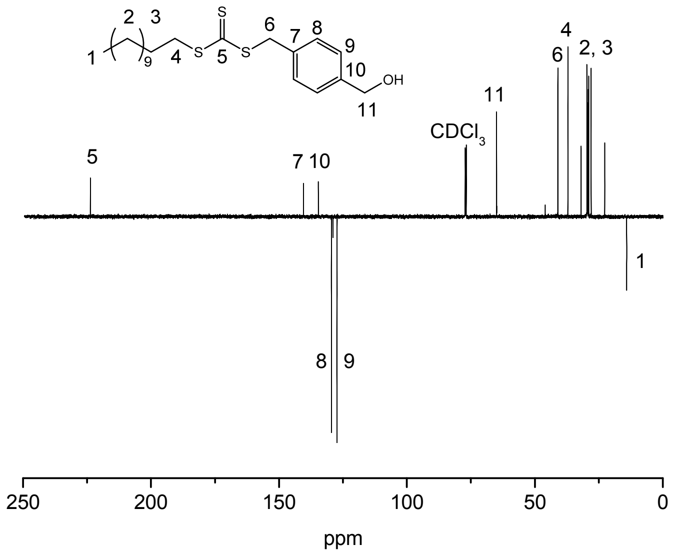

Supplementary Figure 2. ^{13}C NMR spectrum of dual-headed initiator dodecyl 4-(hydroxymethyl) benzyl carbonotrithioate (150 MHz, $CDCl_3$).

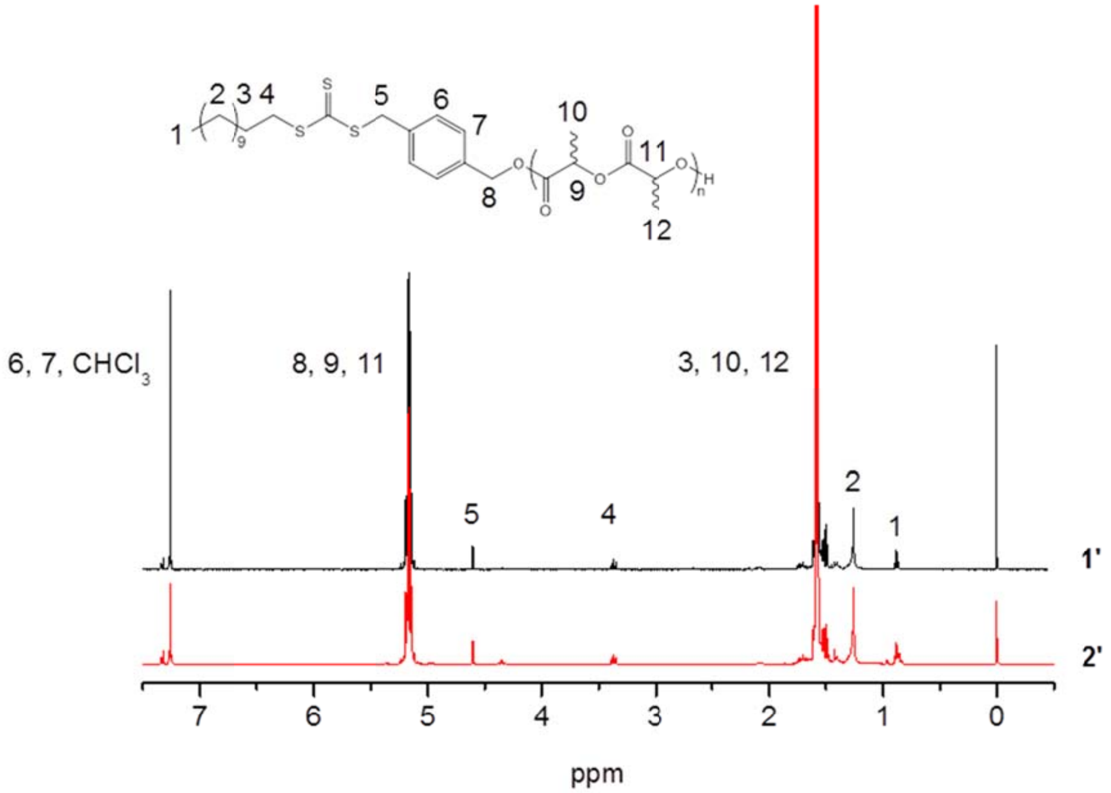

Supplementary Figure 3. ^{1}H NMR spectra of PLLA **1’** and PDLA **2’** (400 MHz, $CDCl_3$).

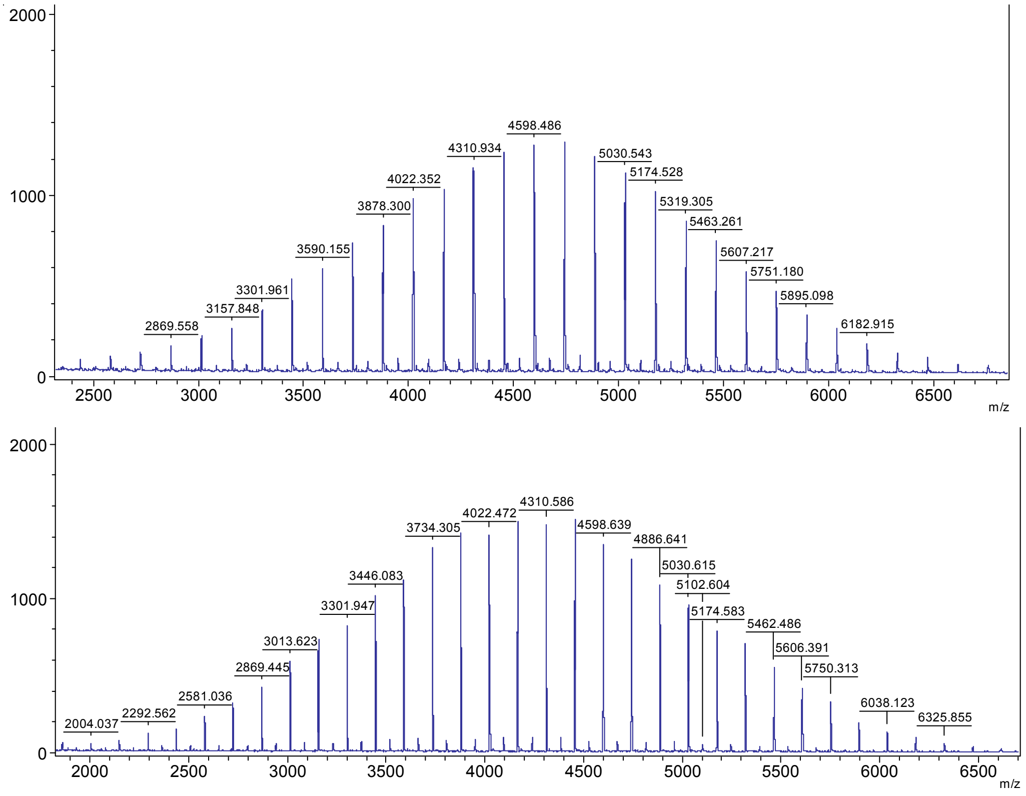

DP	m/z calcu-lated	DP	m/z calcu-lated
11	2005.6282	27	4310.3002
12	2149.6702	28	4454.3422
13	2293.7122	29	4598.3842
14	2437.7542	30	4742.4262
15	2581.7962	31	4886.4682
16	2725.8382	32	5030.5102
17	2869.8802	33	5174.5522
18	3013.9222	34	5318.5942
19	3157.9642	35	5462.6362
20	3302.0062	36	5606.6782
21	3446.0482	37	5750.7202
22	3590.0902	38	5894.7622
23	3734.1322	39	6038.8042
24	3878.1742	40	6182.8462
25	4022.2162	41	6326.8882
26	4166.2582		

Supplementary Figure 4. MALDI-ToF mass spectra of macro-CTAs PLLA **1’** and PDLA **2’**, showing minimal trans-esterification.

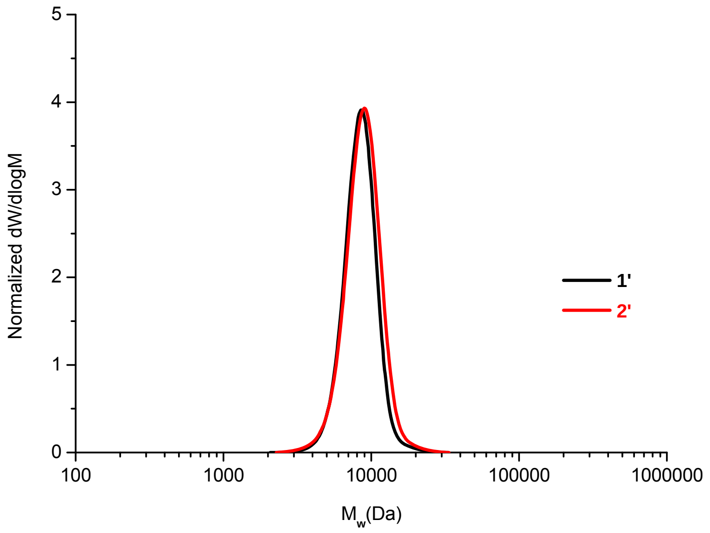

Supplementary Figure 5. SEC traces of PLLA **1’** and PDLA **2’** (THF with 2% TEA as eluent, RI detection).

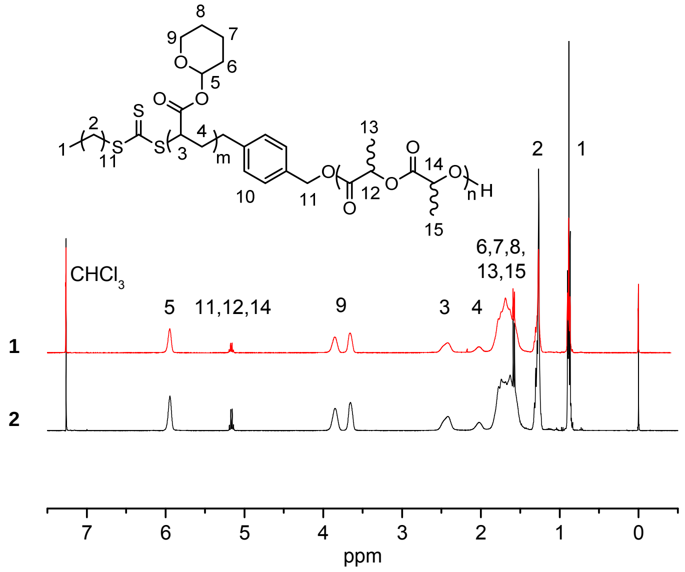

Supplementary Figure 6. ^{1}H NMR spectra of diblock copolymers **1** and **2** (400 MHz, $CDCl_3$).

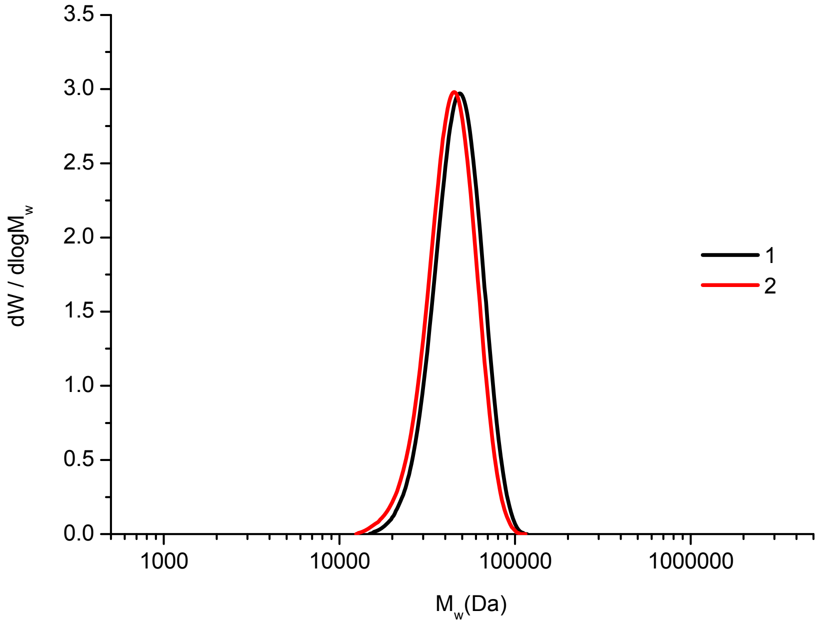

Supplementary Figure 7. SEC traces of **1** and **2** ($CHCl_3$ with 0.5 % TEA as eluent, RI detection).

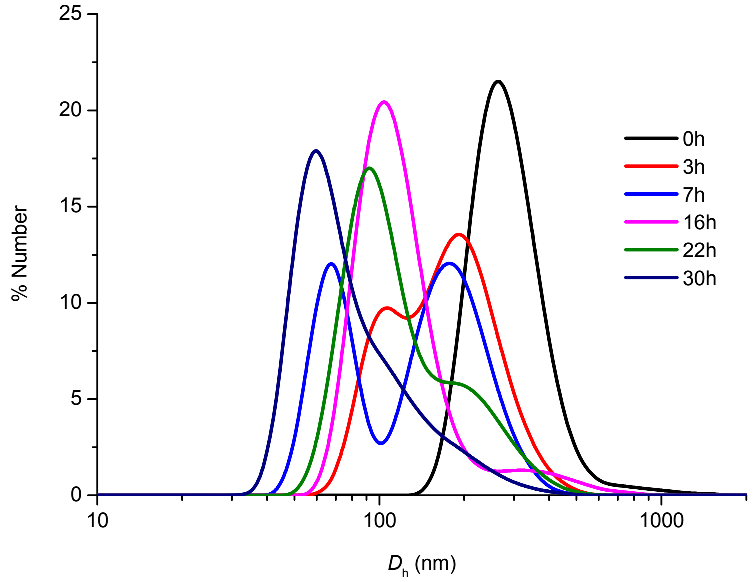

Supplementary Figure 8. DLS data showing that the size of the two homochiral cylinders (**3** and **4** when mixed in a 1:1 ratio with the addition of 20% THF) decreased gradually when mixed together and heated at 65 °C over time.

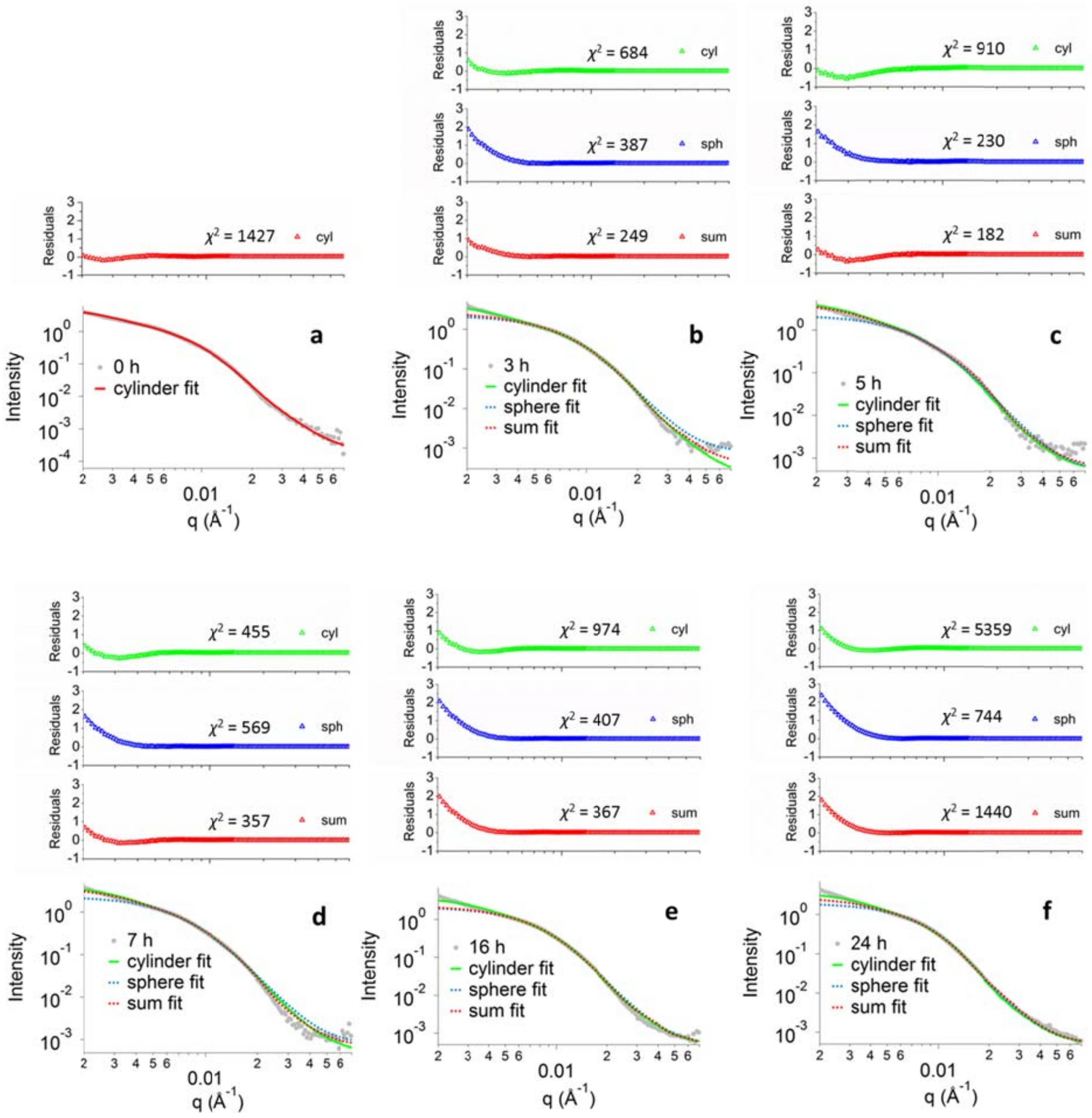

Supplementary Figure 9. SAXS profiles, fits, residuals and χ2 values for the assembly of the mixture of the 2 homochiral cylinders in the presence of THF. Residuals for the sphere model are not well distributed. χ^2 values are better for the sum model than the cylindrical model, which confirms the presence of spherical micelles in the solution.

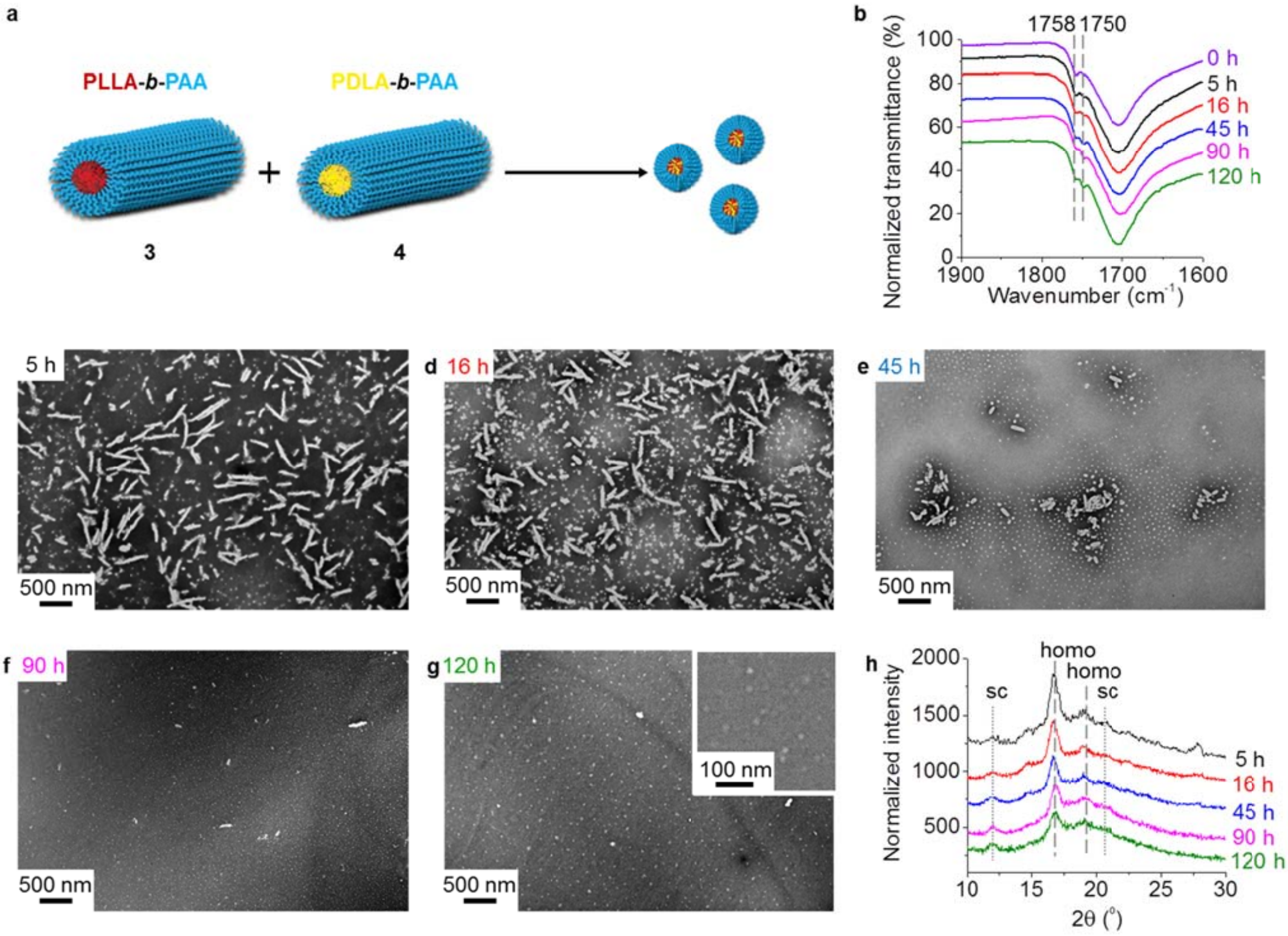

Supplementary Figure 10. Characterization data of the morphological transition and the changes in the crystalline core nature when the two homochiral cylinders 3 and 4 were mixed at 65 °C without the addition of fresh THF. **a**, Cartoon illustrations showing the morphological transition from homochiral cylinders **3** and **4** to stereocomplex spheres. **b**, FT-IR spectra of dried nanoparticles which reveal the wavenumber of carbonyl group vibration of poly(lactide) shifted from 1758 cm^{-1} to 1750 cm^{-1} over time. **c**-**g**, TEM images which illustrate the length of the cylindrical micelles decreased while the population of spherical micelles increased over time. **h**, WAXD diffractograms showed that the intensity of the homochiral Bragg peak at 16.6° decreased gradually while the intensity of stereocomplex Bragg peak at a 2θ of 12° increased slightly. The absence of a good solvent (THF) which hindered the poly(lactide) chain folding and stereocomplexation was proposed to explain the weak intensity of the stereocomplex Bragg peak. TEM samples were prepared by slow drying and negatively stained using PTA.

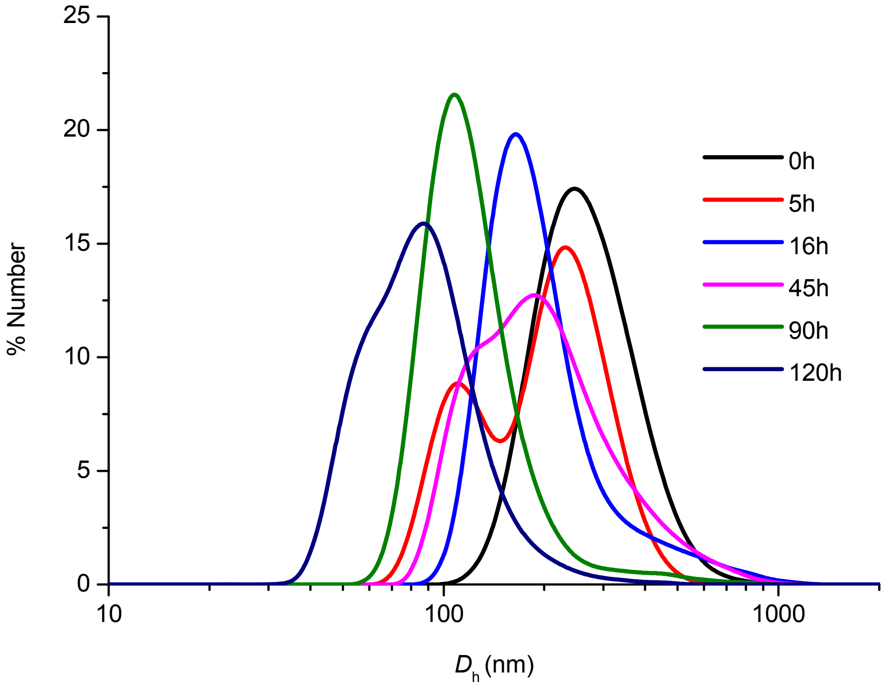

Supplementary Figure 11. DLS data showing that the size of the two homochiral cylinders (**3** and **4** when mixed in a 1:1 ratio without the addition of THF) decreased gradually when mixed together and heated at 65 °C over time.

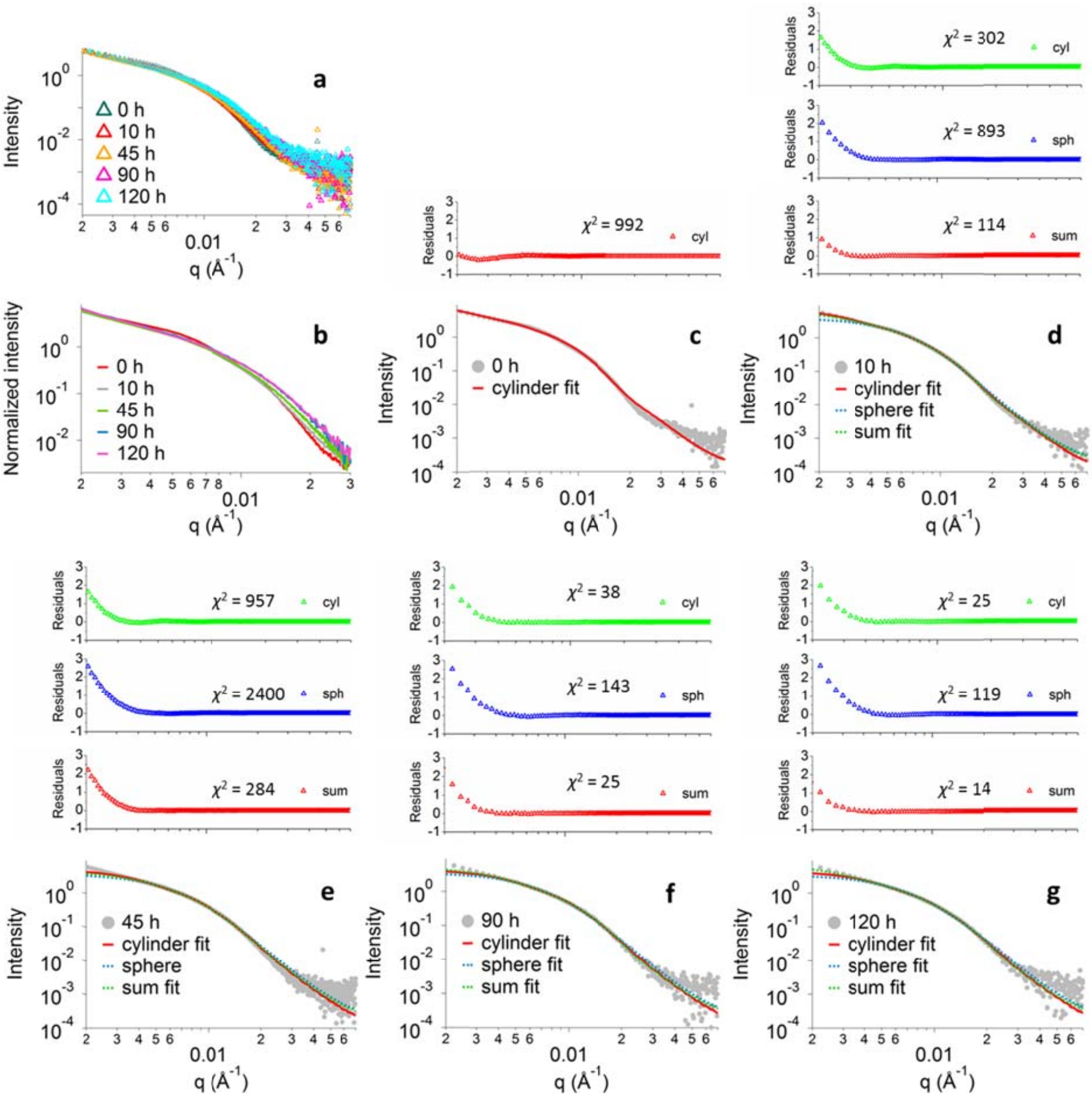

Supplementary Figure 12. SAXS experimental profiles and fittings of the morphological transition from the two homo-chiral cylinders to stereocomplex spheres at 65 °C without the addition of fresh THF at the beginning of the self-assembly. **a**, SAXS experimental profiles during the entire self-assembly process. **b**, A zoom-in figure in the q range of 0.002 to 0.03 Å^{-1}. **c**-**g**, Fittings of experimental profiles by using models "cylinder polyradius", "poly core" and a linear combination of these two models. The fitted parameters are given in Supplementary Table 3.

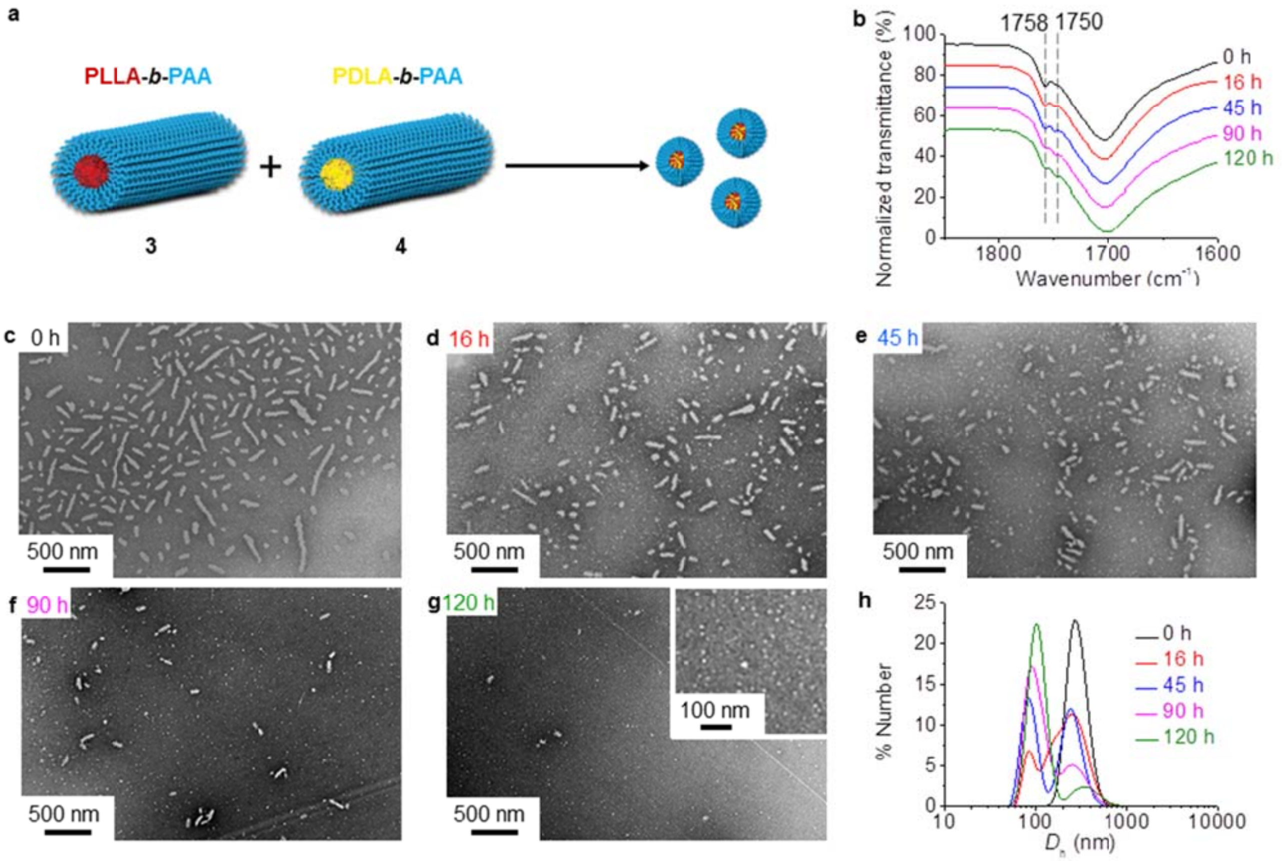

Supplementary Figure 13. Characterization data of the morphological transition when the two homochiral cylinders 3 and 4 (following freeze-dring to remove residual THF) were mixed at 65 °C in nanopure water without the addition of fresh THF. (**a**) Cartoon illustration showing the morphological transition from homochiral cylinders **3** and **4** to stereo-complex spheres. (**b**) FT-IR spectra of dried nanoparticles which reveal the wavenumber of carbonyl group vibration of poly(lactide) shifted from 1758 to 1750 cm^{-1} over time. (**c-g**) TEM images which illustrate the length of the cylindrical micelles decreased while the population of spherical micelles increased over time. (**h**) DLS analysis confirmed the hy-drodynamic diameter decreased during the morphological transition. TEM samples were prepared by slow drying and negatively stained using PTA.

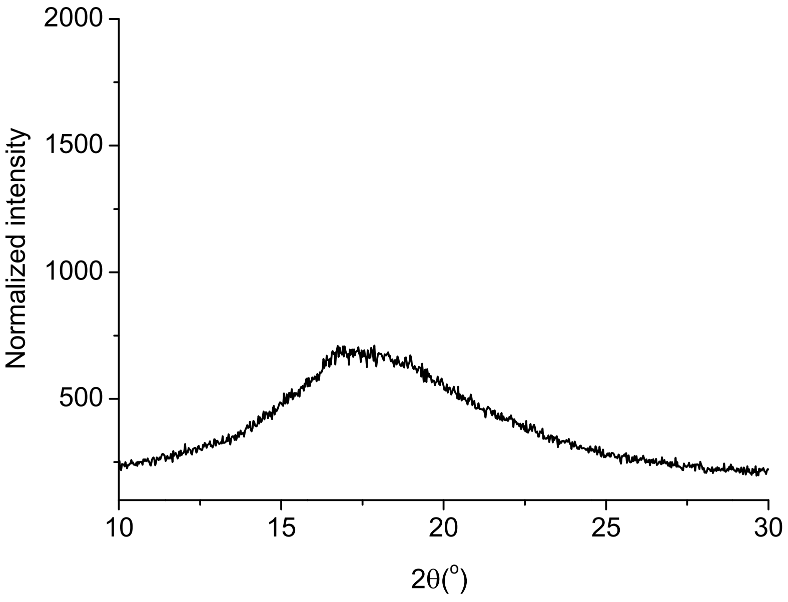

Supplementary Figure 14. WAXD diffractogram showing the amorphous nature of spherical micelles obtained from the self-assembly of **1**.

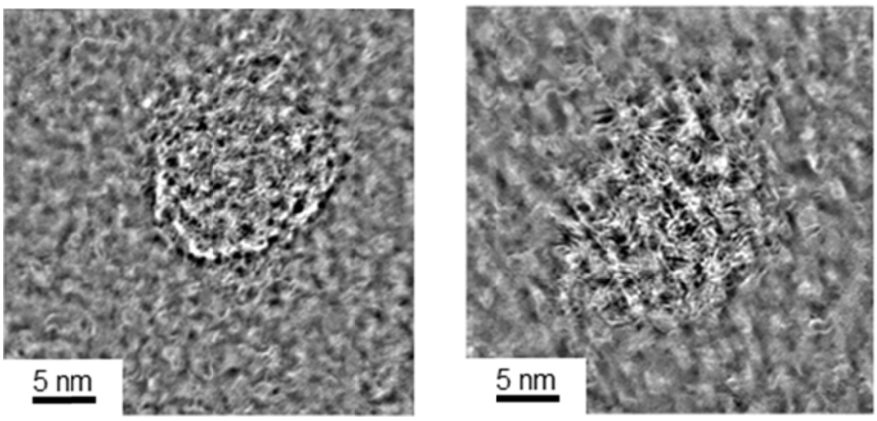

Supplementary Figure 15. EWR phase image of a stereocomplex spherical micelle (**a**, obtained from the mixture of homochiral cylinders **3** and **4**) and an amorphous spherical micelle (**b**, obtained from the self-assembly of **1**) respectively.

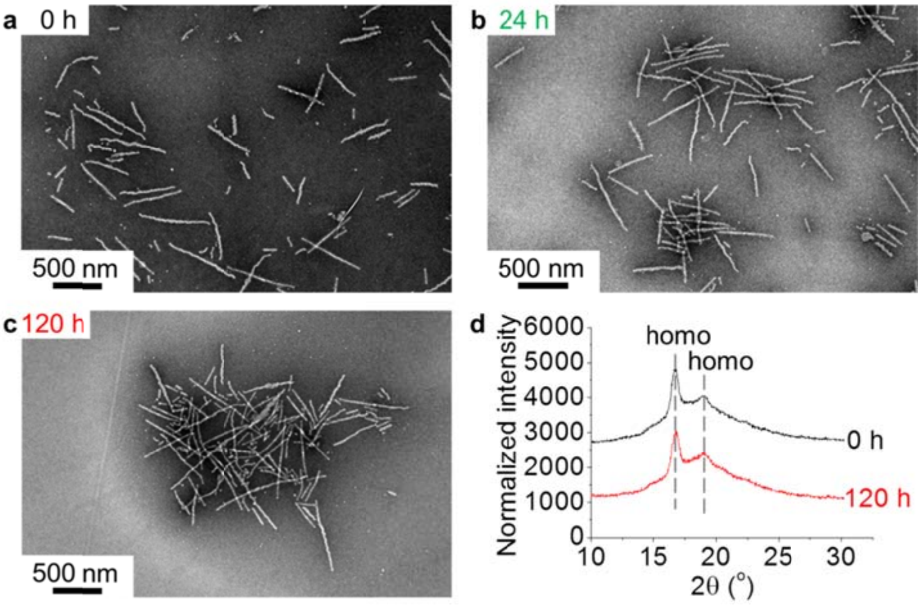

Supplementary Figure 16. a-c, TEM images showing that the lengths of homochiral cylinders 3 did not change significantly when heated at 65 °C for 120 h. d, WAXD diffractograms showing there is no significant difference in the intensity of the homochiral Bragg peak at a 2θ value of 16.6° over this time. No stereocomplex Bragg peaks were observed during the entire self-assembly. TEM samples were prepared by slow drying and negatively stained using PTA.

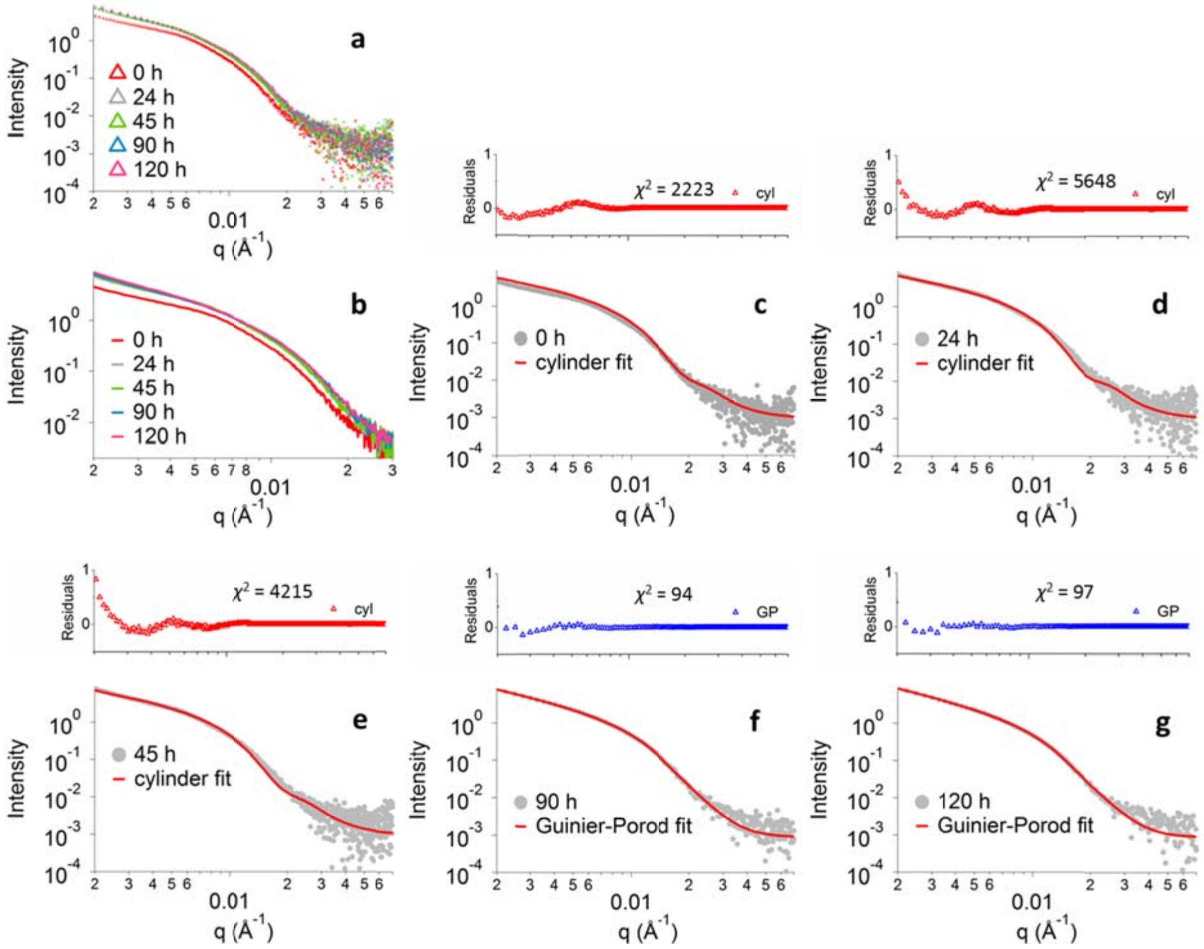

Supplementary Figure 17. SAXS profiles and fittings showing that the size of cylindrical micelles 3 did not change obviously over heating. **a**, SAXS experimental profiles during the entire self-assembly process. **b**, A zoom-in figure in the q range of 0.002 to 0.03 $Å^{-1}$. **c**-**e**, Fittings of experimental profiles by using the cylindrical model. **f-g** Fittings of experimental profiles by using the Guinier-Porod model; some aggregations of the particles were observed and the cylindrical model does not provide correct fits for 90 and 120 h. The parameters of the fitted curves are listed in Supplementary Table 4.

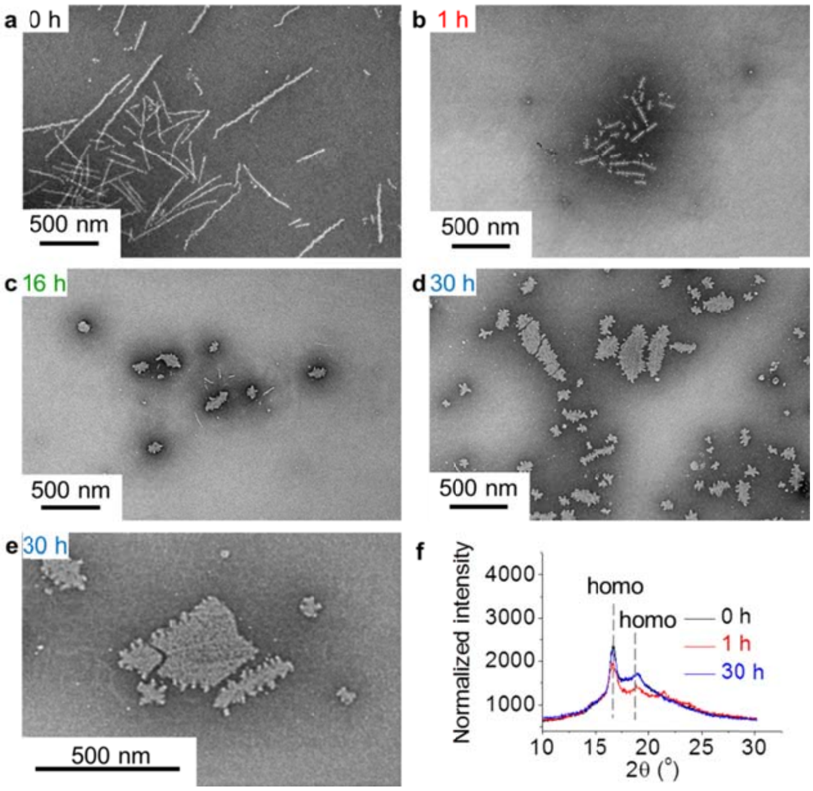

Supplementary Figure 18. TEM images showing that the structure of cylinders 3 changed significantly over time, with heating at 65 °C and with the addition of fresh THF: **a**, Cylindrical micelles before addition of fresh THF. **b**, The length of cylinders decreased dramatically due to dissolution in the THF/water mixture. **c** and **d**, The disassembled unimers gradually inserted into the undestroyed cylinders and lamellar structures were formed;.**e**, Numerous small "arms" were noticed protruding the lamellar micelles. **f**, Overlaid WAXD diffractograms showing that the intensity of homochiral Bragg peak at a 2θ value of 16.6° decreased after addition of fresh THF but recovered after 30 h due to the unimer growth onto the short cylindrical seeds. No stereocomplexation peaks were observed during the self-assembly. TEM samples were prepared by slow drying and negatively stained by PTA.

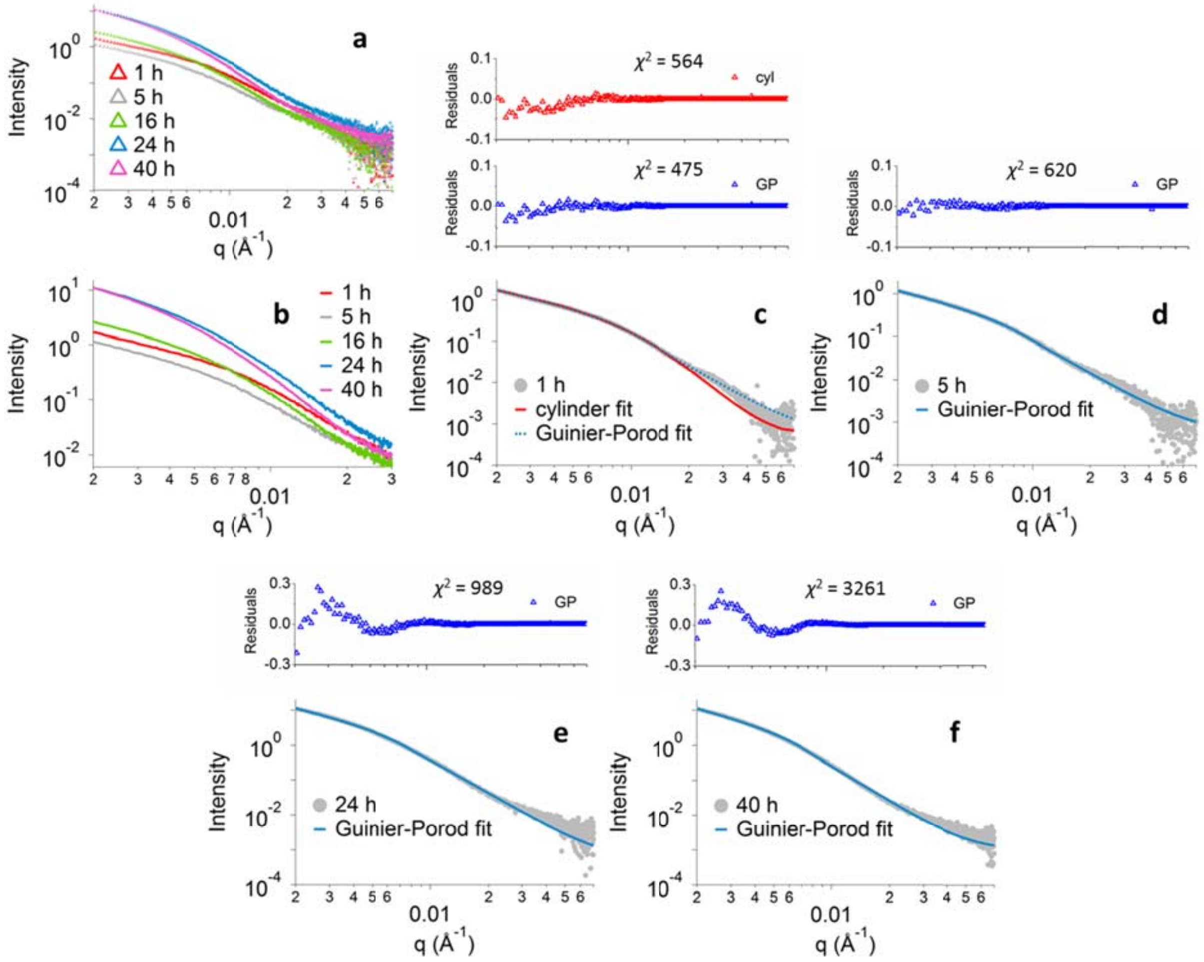

Supplementary Figure 19. SAXS profiles and fittings showing that the structure of cylinders 3 changed significantly into lamellar particles over time after addition of fresh THF and heating at 65 °C. **a**, SAXS experimental profiles during the entire self-assembly process. **b**, A zoom-in figure in the q range of 0.002 to 0.03 $Å^{-1}$. **c**, Fittings of experimental profile by using the cylindrical model and the Guinier-Porod model. **d-f** Fittings of experimental profiles by using the Guinier-Porod model to provide basic information on the general shape of the assemblies. The cylindrical model does not provide a good fit after 5 hrs of heating. The parameters of the fitted curves are listed in Supplentary Table 5.

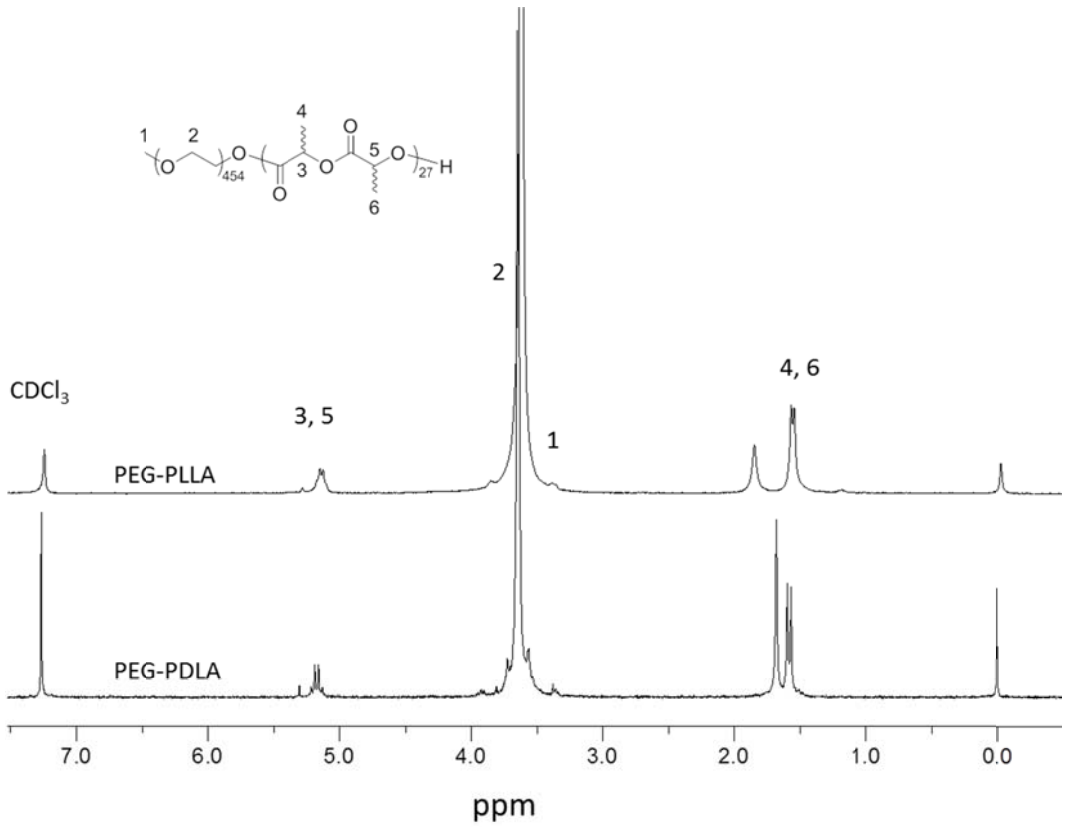

Supplementary Figure 20. ^{1}H NMR spectrum of PEG-*b*-PDLA and PEG-*b*-PLLA copolymers (300 MHz, $CDCl_3$).

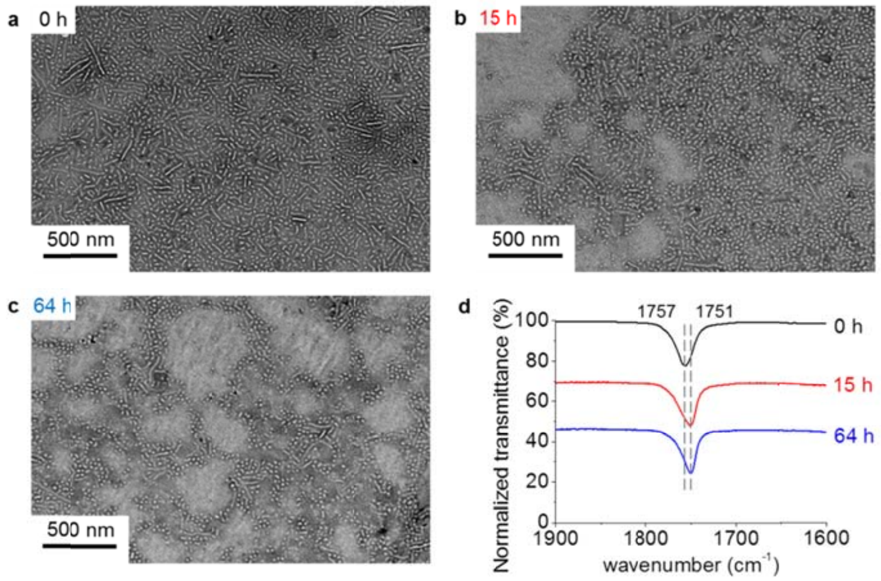

Supplementary Figure 21. Characterization data of the morphological transition and the changes in the crystalline core nature when the two homochiral PLLA-b-PEO cylinder and PDLA-b-PEO cylinder were mixed at 65 °C with the addition of fresh THF. **a**-**c**, TEM images which illustrate the length of the cylindrical micelles decreased while the population of spherical micelles increased over time. **d**, FT-IR spectra of dried nanoparticles which reveal the wavenumber of carbonyl group vibration of poly(lactide) shifted from 1757 cm^{-1} to 1751 cm^{-1} over time. TEM samples were prepared by slow drying and negatively stained by PTA.

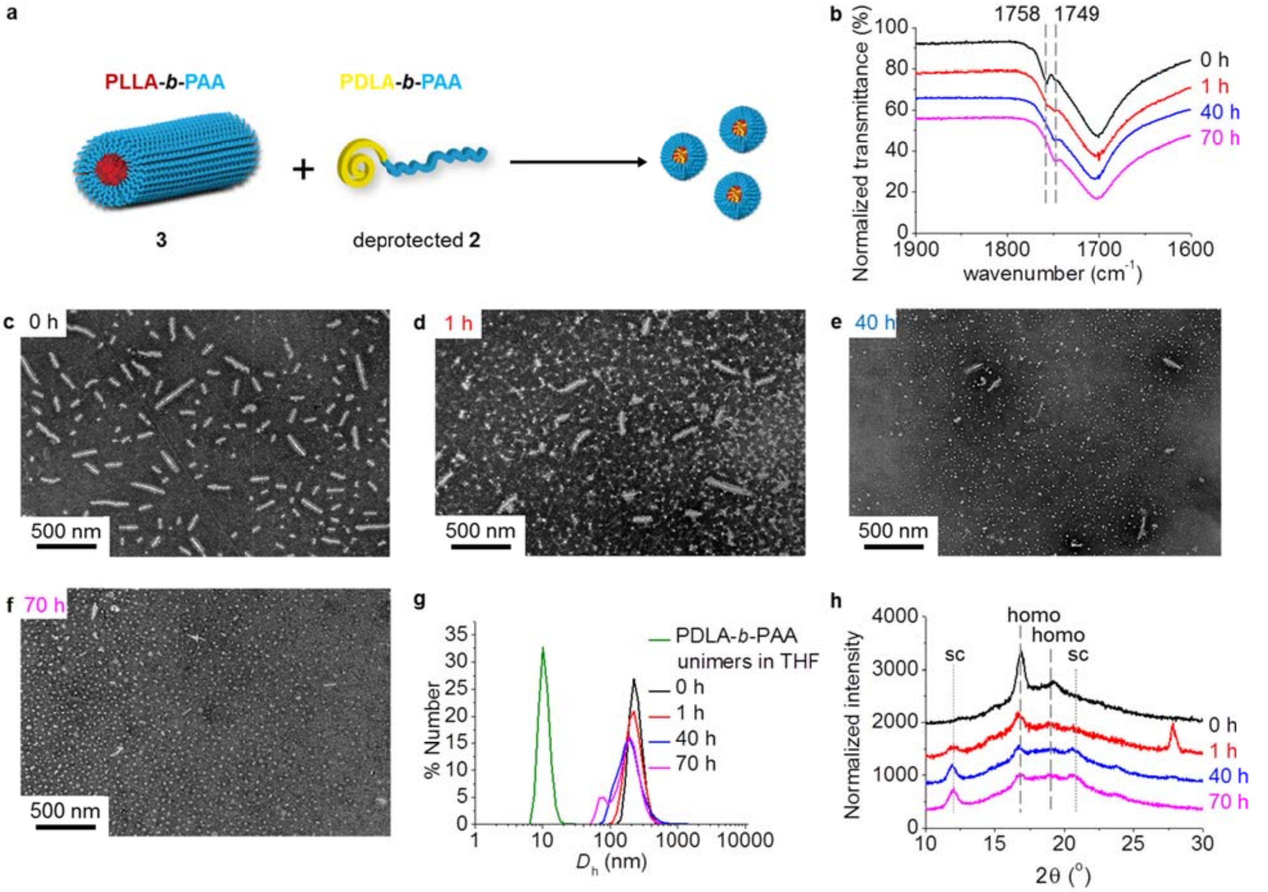

Supplementary Figure 22. Characterization data of the morphological transition and the changes in the crystalline core nature when the THF solution of PDLA-b-PAA unimers was added into aqueous solution of PLLA-b-PAA cylinder 3 at 65 °C. **a**, Cartoon illustrations showing the morphological transition. **b**, FT-IR spectra of dried nanoparticles which reveal the wavenumber of carbonyl group vibration of poly(lactide) shifted from 1758 cm^{-1} to 1749 cm^{-1} over time. **c**-**f**, TEM images which illustrate the length of the cylindrical micelles decreased while the population of spherical micelles increased over time. **g**, DLS data which indicate the morphological transition over time. **h**, WAXD diffractograms showed that the intensity of the homochiral Bragg peak at a 2θ value of 16.6° decreased significantly while the intensity of stereocomplex Bragg peak at a 2θ value of 12° increased gradually. TEM samples were prepared by slow drying and negatively stained using PTA.

Supplementary Table 1. Characterization data of various poly(lactide) macro-CTAs and diblock copolymers

Polymer	M_n (kDa)[a]	$Đ_M$	Hydrophobic weight fraction (%)[d]
$PLLA_{31}$ **1'**	4.7	1.07[b]	—
$PDLA_{28}$ **2'**	4.3	1.08[b]	—
$PLLA_{31}$-*b*-$PTHPA_{332}$ **1**	54.9	1.10[c]	15.7
$PDLA_{28}$-*b*-$PTHPA_{315}$ **2**	52.0	1.11[c]	15.1

[a] Measured by ^{1}H NMR spectroscopy (400 MHz, $CDCl_3$). [b] Measured by THF SEC. [c] Measured by $CHCl_3$ SEC. [d] PLA weight fraction in the final PLA-*b*-PAA diblock copolymer.

Supplementary Table 2. Detailed analysis of the fitted SAXS profiles given in Figure 4 for the assembly of the mixture of the 2 homochiral cylinders. The initial time point experiment profile (t = 0 h) has been fitted with a cylinder model, all the other subsequent profiles with the sum model[a]

time (h)	length of cylinder (nm)	radius of cylinder (nm)	radius of sphere (nm)	scale ratio of cylin-der:sphere[b]
0	191 ± 2	13 ± 0.1	-	-
3	169 ± 1	13 ± 3	12 ± 6	1:0.25
5	150 ± 5	25 ± 25	13 ± 4	1:5.75
7	146 ± 2	25 ± 4	14 ± 2	1:5
16	131 ± 10	13 ± 3	13 ± 2	1:120
24	129 ± 1	20 ± 2	12 ± 1	1: 33

[a] These data have been fitted with cylindrical, sphere and sum models. While the cylindrical model showed a decrease of length of the cylinders over time fits were good at low q but not as good at q higher than 0.011 Å^{-1} which suggested the presence of another morphology in the solution. Analysis of the data using a spherical fit did not provide a good correlation at low q but did provide better correlation at high q. Thus a sum model was created to better model the system through the transition from cylinder to sphere. The better chi square values (data not shown) for the sum model (over either cylinder or sphere model alone) indicate a better fit overall to this model. [b] The scale ratio given by the sum model cannot be used as a ratio between the number of particles of each morphologies. It is related to the volume fraction of each morphology, and thus it is possible to observe trends in the evolution of the comparison of these two numbers.

Supplementary Table 3. Different parameters of the fitted SAXS profiles given in Supplementary Figure 12. The initial time point experiment profile (t = 0 h) has been fitted with a cylinder model, all the other ones with the sum model

time (h)	length of cylinder (nm)	radius of cyl-inder (nm)	radius of sphere (nm)	scale ratio of cylin-der:sphere[a]
0	190 ± 2	17 ± 1	—	—
5	183 ± 2	17 ± 0	19.2 ± 0.2	1:0.7
16	193 ± 7	17 ± 0	18.2 ± 0.7	1:0.8
45	147 ± 1	17 ± 0	16.5 ± 0.2	1:3.1
90	128 ± 6	17 ± 0	14.8 ± 0.5	1:3.8
120	62 ± 4	17 ± 0	14.0 ± 0.7	1:12.5

[a] The scale ratio given by the sum model cannot be used as a ratio between the number of particles of each morphologies. It is related to the volume fraction of each morphology, and thus it is possible to observe trends in the evolution of the comparison of these two numbers.

Supplementary Figure 4. Different parameters of the fitted SAXS profiles given in Supplementary Figure 17 using a "cylinder polyradius" model. As fits were not valuable after 90 h, a simpler model "Guinier-Porod" was used to provide information on the general shape of the assemblies

time (h)	length of cylinder (nm)	radius of cylinder (nm)	dimensionality parameter[a]	shape[a]
0	255 ± 4	19 ± 0.0	2.16	Rod
5	254 ± 5	19 ± 0.0	2.09	Rod
10	275 ± 2	19 ± 0.0	2.09	Rod
16	256 ± 4	19 ± 0.0	2.05	Rod
24	272 ± 3	19 ± 0.0	1.96	Rod
45	277 ± 4	19 ± 0.0	1.94	Rod
63	240 ± 3	19 ± 0.0	1.86	Rod
90	1853 ± 69	19 ± 0.0	1.81	Rod+plate
120	2028 ± 65	19 ± 0.0	1.77	Rod+plate

[a] The dimensionality parameter is calculated as 3-s and is 3 for spherical objects, 2 for rods and 1 for plates. These values are obtained with a Guinier-Porod model.

Supplementary Table 5. Different parameters of the fitted SAXS profiles given in Supplementary Figure 19 using the Guinier-Porod model

time (h)	R_g (nm)	dimensionality parameter[a]	shape[a]
0	16 ± 0.1	2.12	Rod
1	12 ± 0.1	1.97	Rod
5	15 ± 0.1	1.88	Rod+Plate
16	15 ± 0.1	1.81	Rod+Plate
24	16 ± 0.1	1.70	Rod+Plate
40	17 ± 0.1	1.60	Rod+Plate

[a] The dimensionality parameter is calculated as 3-s and is 3 for spherical objects, 2 for rods and 1 for plates.
